# *Guazuma ulmifolia* butanol extract protects against cadmium-induced hepatotoxicity via HO-1/Sirt-1 activation, miRNA–lncRNA modulation, and metabolic reprogramming

**DOI:** 10.1371/journal.pone.0355464

**Published:** 2026-08-06

**Authors:** Osama Ahmed Elsabagh, Abdelbaset M. Elgamal, Heba A. Hassan, Naglaa M. Ammar, Zeinab A. El-Gendy, Mohamed A. El-Saied, Mawada Mohamed Ali, Reham S. Ibrahim, Ahmed H. El-Desoky, Emad M. Hassan, Mohamed A. Farag, Abdelsamed I. Elshamy

**Affiliations:** 1 Pharmacognosy Department, College of Pharmaceutical Sciences and Drug Manufacturing, Misr University for Science and Technology (MUST), Cairo, Egypt; 2 Department of Chemistry of Microbial and Natural Products, Pharmaceutical and Drugs Research Institute, National Research Centre, Giza, Egypt; 3 Therapeutic Chemistry Department, National Research Centre, Dokki, Giza, Egypt; 4 Pharmacology Department, Medical Research and Clinical Studies Institute, National Research Centre, Dokki, Giza, Egypt; 5 Department of Pathology, Faculty of Veterinary Medicine, Cairo University, Giza, Egypt; 6 Department of Pathology, Faculty of Veterinary Medicine, King Salman International University (KSIU); 7 Department of Pharmacognosy, Faculty of Pharmacy, Alexandria University, Egypt; 8 Pharmacognosy Department, National Research Center, Dokki, Giza, Egypt; 9 Medicinal and Aromatic Plants Research Department, National Research Centre, Dokki, Giza, Egypt; 10 Pharmacognosy Department, Faculty of Pharmacy, Cairo University, Cairo, Egypt; 11 Department of Natural Compounds Chemistry, National Research Centre, Dokki, Giza, Egypt; Jadavpur University, INDIA

## Abstract

**Background:**

*Guazuma ulmifolia* is traditionally used for liver disorders, but its protective mechanisms against heavy metal toxicity are poorly defined. This study evaluated the phytochemical profile and hepatoprotective mechanisms of *G. ulmifolia* butanol extract (Gul-BuOH) against cadmium-induced liver injury.

**Methods and findings:**

Gul-BuOH was chemically profiled by UPLC-PDA-ESI–qTOF-MS/MS. Cadmium hepatotoxicity was induced in rats, followed by Gul-BuOH treatment (100 and 200 mg/kg). Liver injury, oxidative stress, inflammation, gene expression (Let-7a, HOTAIR), histopathology, HO-1/Sirt-1 immunoreactivity, and serum metabolomic changes were assessed. Chemical profiling led to the annotation of 42 compounds, including mainly flavonoids and phenolic acids, highlighting the rich phytochemical composition of *G. ulmifolia*. CdCl_2_ exposure increased hepatic Cd accumulation and elevated ALT, AST, and ALP, reduced TAC, and increased NO and MDA. Gul-BuOH significantly reduced hepatic Cd levels by 2.6- and 3.2-fold, restored TAC by 38.3% and 83.2%, and decreased NO (56.3% and 63.4%) and MDA (45.4% and 54.6%) at 100 and 200 mg/kg, respectively. Inflammatory markers NF-κB-p and TNF-α were markedly suppressed, while miRNA Let-7a was upregulated and lncRNA HOTAIR was downregulated. Histological and immunohistochemical analyses revealed near-complete restoration of hepatic architecture and normalization of HO-1 and Sirt-1 expression at the high dose. Serum metabolomics’ OPLS-DA model performance indicators demonstrated strong reliability, with an R^2^Y (explained variance) of 0.991 and a Q^2^ (predictive variance) of 0.988). The model identified 36 significantly altered metabolites that were largely normalized by Gul-BuOH, implicating linoleic acid metabolism, amino acid biosynthesis, and ascorbate-related pathways.

**Conclusion:**

Gul-BuOH affords dose-dependent protection against Cd-induced liver injury by modulating oxidative stress, inflammation, metal detoxification, and metabolic pathways, supporting the traditional use of G. ulmifolia and its potential as a multi-target hepatoprotective agent.

## 1. Introduction

The liver is a key metabolic organ vulnerable to systemic changes and toxicant exposure. Hepatic malignancies rank as the second most prevalent cancer globally, linked to significant mortality [1]. Environmental toxicants, especially heavy metals, including cadmium (Cd), pose serious health risks, with effects dependent on exposure route, age, nutrition, and genetics [2]. Over 90% of Cd contamination arises from human activities, with annual industrial production at 13,000 tons for batteries, coatings, and fertilizers [3,4]. Cd bioaccumulates significantly in biological systems, mainly affecting the liver and kidneys, which together store about half of the body’s Cd burden, with additional deposits in bones and endocrine organs [3].

Human exposure to Cd occurs mainly through gastrointestinal absorption, inhalation, and, to a lesser extent, dermal contact [5]. Human exposure to Cd occurs mainly through gastrointestinal absorption, inhalation, and, to a lesser extent, dermal contact [5]. Even low environmental levels are linked to skeletal demineralization and systemic disorders [3]. Occupational limits, such as the German threshold of 15 μg/L, highlight its toxicity compared to the average blood concentration of 0.5 μg/L in nonsmokers [6]. The rise in Cd pollution, driven by industrial activities, presents significant ecological and health risks [7]. Cd toxicity is associated with oxidative stress, where imbalances lead to DNA, lipid, and protein damage, significantly contributing to hepatic disorders, including fibrosis and hepatocellular carcinoma [8,9].

Medicinal plants are abundant in antioxidant phytochemicals, especially flavonoids and phenolic compounds, which can combat free radicals and mitigate oxidative stress [10,11]. *Guazuma ulmifolia* Lam., known as Mutamba, is traditionally used in Latin America and the Caribbean for treating various ailments, including gastrointestinal and respiratory disorders, fever, and liver diseases [12–14]. In Peru, decoctions of its bark and leaves serve as antidiarrheal and anti-inflammatory remedies, with its fruits used for soothing sore throats [15]. Phytochemical studies reveal that *G. ulmifolia* contains bioactive compounds such as flavonoids and saponins, which contribute to its medicinal properties [16,17]. Pharmacological research indicates its broad biological activities, including antioxidant, anti-inflammatory, and cytoprotective effects, supporting its traditional uses and potential as a therapeutic agent [16–20].

MicroRNAs (miRNAs) are non-coding RNAs, 20–24 nucleotides long, that regulate gene expression post-transcriptionally. They are transcribed as primary miRNAs in the nucleus, processed, and exported to the cytoplasm, where they mediate gene silencing [21]. Specifically, miR-let7a has anti-inflammatory effects by repressing target genes and modulating signaling pathways; its dysregulation can hinder cellular differentiation and contribute to diseases [22]. Long non-coding RNAs (lncRNAs), greater than 200 nucleotides and non-protein coding, also play significant roles in regulatory processes and are now recognized for their importance in cellular functions, contrary to their prior perception as merely transcriptional noise [23].

Cadmium exposure significantly disrupts the non-coding RNA regulatory network in hepatocytes, particularly affecting HOTAIR, a stress-responsive lncRNA that sequesters tumor-suppressive microRNAs like Let-7a. Using qRT-PCR, researchers quantified the genetic and epigenetic changes in hepatic tissue due to cadmium [23]. Additionally, Sirt1, a key regulator in the hepatic response to oxidative stress, is suppressed by cadmium, impairing the antioxidant response mediated by Heme Oxygenase-1 (HO-1) and leading to hepatocellular apoptosis. Immunohistochemical analysis of Sirt1 and HO-1 facilitated the assessment of their expression levels and spatial distribution in the injured liver tissue [22,23].

This study aimed to (i) comprehensively characterize the phytochemical profile of *G. ulmifolia* leaf butanol extract (Gul-BuOH), (ii) evaluate its hepatoprotective potential against CdCl_2_–induced liver injury, (iii) investigate underlying mechanisms including antioxidant and anti-inflammatory effects, modulation of miRNA Let-7a and lncRNA HOTAIR, histopathological and immunohistochemical alterations, and metabolic pathway regulation *via* serum metabolomics.

## 2. Materials and methods

### 2.1 Plant collection, authentication and preparation

Leaves of *G. ulmifolia* were sourced in June–July 2023 from the Shehab Mazhar Botanic Garden in Giza, Egypt (30.06601, 31.14407), coinciding with the plant’s peak flowering period [24]. Sampling was carried out over multiple early-morning field visits (05:30–06:30) to obtain fresh and representative material. The collected specimens were subsequently verified and authenticated by Dr. Trease Labib, taxonomist at El-Orman Botanic Garden. An herbarium voucher (YrQ-8871-TxGZU-117–2023) was prepared and deposited in the garden’s official collection to serve as a permanent reference for the future aspects.

### 2.2 Plant preparation and extraction

Following collection, the *G. ulmifolia* leaves were first freed of surface impurities and left to dry naturally in a shaded, aerated space for 15 days, ensuring full dehydration. Once dried, the leaves were milled with a sterile mechanical grinder to obtain a fine, uniform powder, producing 570 g of processed plant material. For extraction, the powdered sample was immersed in 4.5 L of a *n*-butanol and allowed to macerate at ambient temperature for one week. The resulting mixture was filtered, and the solvent was gently removed under reduced pressure at 45–50 °C using a rotary evaporator. This procedure yielded 18.5 g of a dense, dark black extract (GU-BuOH). The final extract was transferred to sterile, light-protected glass vials and stored at –4 °C pending further analyses.

### 2.3 UPHPLC-ESI-MS/MS analysis experimental procedure

Sample (2 µL) of extract prepared at a concentration of 1 mg/ml in 100% MeOH was injected via partial injection mode, into a Waters ACQUITY I-Class UHPLC system made up of Binary Solvent Module, FL Sample Manager, UHPLC eLambda 800 nm (wavelength 190–600 nm with resolution 1.2 nm) and separated at a flow rate of 300 µL/min at 55 ℃ on a Waters ACQUITY UHPLC BEH C18 column (50 mm length × 2.1 mm internal diameter, 1.7 µm particle size, Waters GmbH, Eschborn, Germany). Eluting solvents included water (A; ultrapure water from Barnstead™ GenPure™, Thermo Scientific™) and CH_3_CN (B; Chromasolv™, for LC-MS, Honeywell Riedel de Ha ¨ en™) with 0.1% formic acid (additive for LC-MS, LiChropur®, Merck). Chromatographic separation was performed using an optimum elution gradient as follows: solvent B at 3% (isocratic for 1 min) increasing to 95% B (within 7 min), then at 95% B (3 min), followed by re-equilibration of the column at 3% B (2.5 min). The column effluents were online infused in a hybrid qTOF mass spectrometer (Sciex TripleTOF 6600 LC-MS System, AB Sciex, Darmstadt, Germany) run in negative ion mode. The ion spray voltage was set to 4500 V, source temperature 450 ℃, while the nebulizer, drying, and curtain gases were set to 85, 70, and 55 psig, respectively. The MS experiments were performed in an *m/z* range of 50–1500 in the TOF-scan mode (accumulation time 100 ms). The MS2 experiments in information dependent acquisition (IDA) mode (*m/z* 50–1500, mass tolerance 25 ppm, intensity ˃ 100, exclude isotope window 4 Da) were accomplished with 50 ms accumulation time at the collision potential (CE) of −40 V, with collision energy spread (CES) of 10 V and declustering potential. (DP) of −35 V. Nitrogen was employed as the collision activation dissociation (CAD) gas. The identification of metabolites relied on several criteria: order of elution, ultraviolet-visible (UV/Vis) spectroscopy, molecular formulas predicted using high-resolution mass spectrometry (HR-MS), fragmentation patterns in tandem MS-MS, matching with known standards, and comparison against a proprietary database and Mass Bank (https://massbank.jp/) as well as the Dictionary of Natural Products (DNP, 2015) [25,26].

### 2.4 *In vivo* biological assessments

#### 2.4.1 Chemicals and reagents.

Cadmium chloride (CdCl_2_, 99.99% trace metals basis; CAS No. 10108-64-2) was purchased from Sigma-Aldrich (USA). All other chemicals and reagents used were of analytical grade.

#### 2.4.2 Animals and ethical statement.

A cohort of twenty-four adult male Wistar albino rats, weighing 180–200 g, was procured from the animal breeding facility of the National Research Center (NRC, Giza, Egypt). The rats were maintained under standardized laboratory conditions, including controlled ambient temperature (25 °C), relative humidity (≈50%), and a 12 h light/12 h dark photoperiod. Prior to experimental manipulation, all rats were allowed a 7-day acclimatization period. Standard laboratory chow and potable water were provided ad libitum throughout the study period. All experimental protocols were designed and conducted in strict accordance with internationally accepted ethical standards for animal experimentation, including the guidelines of the Institutional Animal Care and Use Committee (IACUC) and Faculty of Veterinary medicine, Cairo University the National Institutes of Health (NIH publication No. 85–23, revised 2011). Ethical approval was obtained from the Ethics Committee of the National Research Center, Egypt (approval no. **Vet CU 25122023834**), and the study was reported in compliance with the ARRIVE guidelines. Comprehensive measures were implemented throughout the experimental period to minimize rats’ distress, prevent unnecessary suffering, and avoid significant body weight loss.

#### 2.4.3 Experimental protocol.

**Fig 1:**
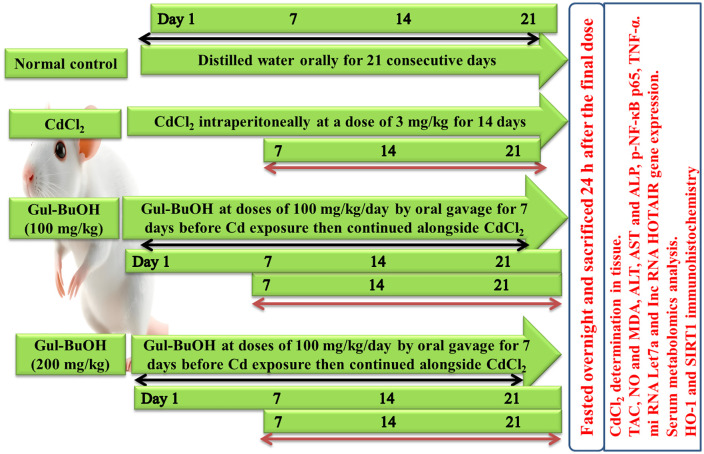
Schematic illustration of the experimental design timeline showing the pretreatment and co-treatment phases, timing of tissue collection, and the assessed experimental parameters.

Based on earlier toxicological evaluations demonstrating the safety profile of *G. ulmifolia* leaves that demonstrated [12], the plant extract was considered suitable for *in vivo* assessment. Following a 7-day acclimation period, the twenty-four rats were randomly allocated into the four experimental groups (6 rats per group) using a body weight randomization method to minimize selection and allocation bias. The control cohort (Group I) received distilled water orally for 21 consecutive days. In the cadmium-intoxicated cohort (Group II), rats were administered CdCl_2_ intraperitoneally at a dose of 3 mg/kg for 14 days; this sublethal dose was selected according to previously published toxicological data [27]. In the treatment cohorts (Groups III and IV), rats were pretreated with the Gul-BuOH at doses of 100 and 200 mg/kg/day, respectively, by oral gavage for 7 days before cadmium exposure. Thereafter, Gul-BuOH administration was continued alongside CdCl_2_ (3 mg/kg, i.p.) for an additional 14 days. During the co-exposure period, Gul-BuOH was given orally, followed one hour later by intraperitoneal injection of CdCl_2_ (Fig 1). All dosing solutions were freshly prepared in distilled water at the required concentrations prior to administration. Throughout the experimental duration, rats were closely monitored, and no mortality was observed in any group.

Overall rats were observed every day during the evaluation period for clinical indicators of distress, illness, or death, such as alterations in body weight, grooming habits, food and water consumption, posture, and locomotor activity. In compliance with institutional animal care norms and the authorized ethical protocol, any rat displaying severe or persistent signs of pain or discomfort was promptly euthanized. Humane endpoints were established to reduce suffering. The rat welfare was the primary concern from the beginning of the investigation until its ending. Whenever possible, a number of proactive and compassionate steps were taken to prevent, lessen, or completely eradicate pain and suffering. As the study progressed, each rat was intensively observed, first once a day and later twice a day. These welfare inspections were carried out by a qualified laboratory animal technician, guaranteeing expert supervision. Key parameters, including breathing effort and rate, were meticulously measured and revealed a range from muted to normal. Behavior, posture, and movement were also observed, creating a complete picture of each rat’s condition. Daily records of each rat’s body weight provided crucial information about stability and health. A Universal Interface Device (UID) reader, which provides accurate and minimally invasive readings, was used once or twice daily to monitor core temperatures. A non-contact infrared thermometer (Lasergrip 774, Etekcity Inc., Anaheim, CA, USA) was used to take last temperature readings before to planned euthanasia in compliance with humane endpoints. To minimize stress during the procedure, each rat was gently restrained while temperature measurements were recorded.

At the end of the experimental period (day 21), the rats were fasted overnight and sacrificed 24 h after the final dose. Retro-orbital blood sampling was performed under general anesthesia induced by using sodium phenobarbital (40–50 mg/kg, i.p) to minimize pain and distress during the procedure. All experimental procedures were conducted in accordance with institutional ethical guidelines for the care and use of laboratory animals. Subsequently, upon completion of the experiment, all rats were quickly and humanely euthanized while under anesthesia with an intraperitoneal injection of lethal dose of (300 mg/kg) of sodium pentobarbital. The rats were subsequently disposed of in compliance with the recommendations established out through the National Research Center’s Safety and Health Committee (NRC). The liver was rapidly excised, rinsed with ice-cold saline, and sectioned into four representative aliquots for downstream analyses. One aliquot was reserved for the quantification of hepatic cadmium content. A second aliquot was processed for biochemical investigations by homogenization in ice-cold 0.05 M phosphate buffer (pH 7.0) to yield a 10% (w/v) tissue homogenate using a Mini-BeadBeater-8 homogenizer (Biospec Products, USA). The homogenates were centrifuged at 10,000 rpm for 20 min at 4 °C (Sigma 2K15, Germany), and the collected supernatants were aliquoted and preserved at −80 °C until analysis. A third aliquot was fixed in 10% neutral buffered formalin for routine histopathological and immunohistochemical examinations, whereas the remaining liver tissue was snap-frozen and stored at −80 °C for subsequent molecular assays.

#### 2.4.4 Cadmium determination in liver tissue.

Liver samples were digested in an acid matrix using a microwave digestion system (Multi-wave PRO, Anton-Paar, Graz, Austria) with 5 mL of 65% HNO₃ and 1 mL of 30% H₂O₂. Cadmium concentrations were quantified by inductively coupled plasma–optical emission spectrometry (ICP-OES, Agilent 5100 Synchronous Vertical Dual View, Serial No. MY15180008). Accuracy and precision of the measurements were ensured using external reference standards (Merck, Darmstadt, Germany), as well as standard reference materials and quality control samples from the National Institute of Standards and Technology (NIST, USA). Results were expressed as ng/g tissue [28].

#### 2.4.5 Biochemical analyses.

2.4.5.1 **Estimation of oxidative stress and antioxidant parameters in serum.** The determination of the antioxidant capacity is performed using Colorimetric Total Antioxidant Capacity method catalogue no. (TA 25 13), Nitric oxide was determined using colorimetric nitric oxide assay catalogue no. (NO 25 33) and Malondialdehyde was determined using colorimetric lipid peroxide method catalogue no. (MD 25 29) of Biodiagnostic Co. www.bio-diagnostic@gmail.com [29–31], respectively.

2.4.5.2 **Estimation of liver function enzymes in serum.** The determination of ALT was performed using colorimetric alanine aminotransferase method catalogue no. (264 001), AST was determined using colorimetric aspartate aminotransferase method catalogue no. (260 001) and ALP was performed using colorimetric Alkaline phosphatase method catalogue no. (AP 10 20) of Biodiagnostic Company (www.bio-diagnostic@gmail.com) [32,33], respectively.

2.4.5.3 **Estimation of liver inflammation indicators.** Hepatic levels of phosphorylated NF-κB p65 (p-NF-κB p65) and tumor necrosis factor-α (TNF-α) were determined using rat ELISA kits BioVision, catalogue no. (K4521-100), Milpitas, CA, USA and BT LAB, catalogue no. (E0764Ra), Zürich, Switzerland according to the manufacturers’ protocols (www.biovision.com) tech@biovision.com.

2.4.5.4 **Quantification of liver mi RNA Let7a and Inc RNA HOTAIR gene expression in liver tissue homogenate.** The total RNA was extracted from liver tissue homogenate using miRNeasy RNA extraction kit (Qiagen, Valencia, CA, USA) following the manufacturer’s protocol. The RNA concentration and purity were determined using the Nano Drop2000 (Thermo Scientific, Waltham, MA, USA) (Table 1). Following the manufacturers’ recommendations, cDNA was synthesized using the RevertAid First Strand cDNA Synthesis kit (Thermo Fisher Scientific, USA) for lncRNAs and the miRCURY LNA RT kit (Qiagen, Valencia, USA) for miRNA. For quantitative PCR, the cDNA samples were then amplified using the miRCURY LNA SYBR Green PCR kit and customized primers (eurofins Genomics, GmbH, Germany) according to the manufacturer’s instructions. Their target specificity was examined using the NCBI primer–BLAST tool. The primers’ sequences are listed in Table 1. GAPDH and U6 were employed as internal controls for normalizing lncRNAs and miRNAs, respectively. Fold change was calculated using the 2 − ΔΔCt formula for relative quantification.

**Table 1:** Primer sequence for the studied target genes.

GENE	Primer Sequence
**miR-let7a**	5’- TGAGGTAGTAGGTTGTATAG −3’ (forward) 5’- GCGAGCACAGAATTAATACGAC (reverse)
**lncRNA HOTAIR**	5’- CCAGAGAACGCTGGAAAAACCTG-3’ (forward) 5’- GAACATGTCTGCGTATCTC −3’(reverse)
**U6**	5′-CTCGCTTCGGCAGCACA-3′ (forward) 5′-AACGCTTCACGAATTTGCGT-3′ (reverse)
**GAPDH**	5’-GAACGGGAAGCTCACTGGCATGGC-3’(forward) 5’-TGAGGTCCACCACCCTGTTGCTG-3(reverse)

2.4.5.5 **Serum metabolomics analysis**

2.4.5.5.1 **Serum Sample Preparation and GC-MS Conditions.** After thawing, 100 μL of serum samples was added to 300 μL of cold acetonitrile and centrifuged at 10,000 rpm for 10 minutes. The supernatant was then dried with nitrogen. Then, 50 μL of methoxylamine hydrochloride (dissolved in 20 mg/mL pyridine) was added, stirred gently, and placed in the oven at 70°C for 1 h. Each sample received 100 μL BSTFA (containing 1%TCMS) and was placed for 1 h in oven at 70°C. Finally, 100 μL was then taken for GG-MS analysis. Metabolites were profiled using gas chromatography (Thermo Scientific Corp., USA) with an ISQ Single Quadrupole Mass Spectrometer as the detector. The chromatographic separation was performed using the previous method provided [34].

2.4.5.5.2 **Serum metabolomics and hepatoprotective pathway analysis.** Verifying their retention indices (RI) against those of n-alkane standards (C7–C40). Prior to spectral matching, peak deconvolution was conducted using the MS-Dial platform (https://systemsomicslab.github.io/compms/msdial/main.html) [34]. Mass signal intensities were then normalized and Pareto-scaled before multivariate analysis. Multivariate modeling was performed in SIMCA-P 14.1 (Umetrics, Umea, Sweden) using orthogonal partial least squares–discriminant analysis (OPLS-DA), which facilitates the identification of important metabolites and classification of sample groups. Variable Importance in Projection (VIP) scores were applied to evaluate each feature’s contribution to the OPLS-DA model, with VIP values above 1.0 indicating significant variables [35]. For univariate analysis, metabolites showing p-values below 0.05 and fold changes (FC) greater than 2.0 or less than 0.5 were considered significant, based on t-tests and fold-change analysis performed using MetaboAnalyst 6.0 (https://www.metaboanalyst.ca/). MetaboAnalyst was also used to conduct pathway analysis using the set of key differential metabolites [36].

2.4.5.6 **Histochemical evaluations**

2.4.5.6.1 **Histopathology.** Liver specimens were obtained to assess the extent of pathological changes among the groups under investigation. Samples were preserved in 10% neutral buffered formalin and subsequently processed through standard procedures utilizing a tissue processor (Histo Core PEARL, Leica, Germany) followed by paraffin wax embedding, sectioned at 5 µm thickness for routine Hematoxylin and eosin (H&E) staining. Leica DM4 B light microscope (Leica, Germany) was used for slide examination 10 fields per group was examined, and Leica DMC 4500 digital camera (Leica, Germany) was used for photo capturing at 200x. The extent of the hepatocellular changes was assessed based on the scoring system used previously [37,38]. The hepatic lesion score was assessed for the following changes congestion, inflammation, apoptosis, degeneration and necrosis as 0 means normal, 1 means mild changes with less than25% of liver affected, 2 means moderate with less than 50% of liver affected changes and 3 means severe changes with more than 50% of liver affected.

2.4.5.6.2 **Immunohistochemistry.** Immunohistochemistry technique was performed for detection of Heme Oxygenase-1 (HO-1) and sirtuin 1 (SIRT1). Sections of liver tissue were conducted on positive charged glass slides. Briefly, the tissue sections were submitted for heat-induced epitope retrieval, followed by peroxidase blockage then incubated with monoclonal antibody against HO-1 and Sirt-1 as a primary antibody anti-Sirt-1 (at a dilution of 1:200, Proteintech, Germany) and anti-HO-1 (at a dilution of 1:100, Santa Cruz, Inc). After washing, HRP-labelled detection kit (BioSB, USA) was used as manufacturer instructions to develop the color. Positive expression was visualized as brown color that measured as area % using Olympus CellSens dimensions software (Olympus, Tokyo, Japan) [39]**.**

2.4.5.7 **Statistical analysis.** The Shapiro-Wilk’s test for normality (p > 0.05) validated the normal distribution of data. One-way analysis of variance (ANOVA) was applied to determine statistical disparity between means. Tukey’s test for multiple comparisons was utilized to evaluate statistical significance across distinct groups in cases of significant F-ratio and post hoc, at p < 0.05 or 0.001 degree of significance. The graphs in this study were created with the Graph Pad Prism software (version 7.0 for Windows, Graph Pad Inc., San Diego, CA).

## 3. Results

### 3.1 UPLC-MS-based metabolites profiling *G. ulmifolia* methanol extract

**Fig 2:**
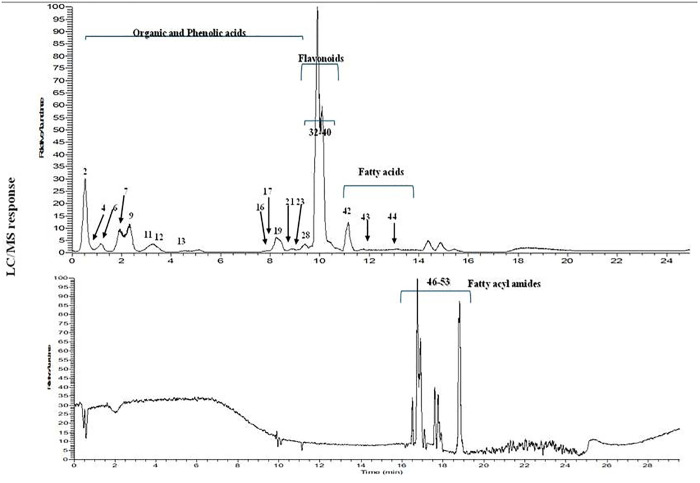
UPLC-PDA-ESI–qTOF-MS/MS ion chromatograms of *G. ulmifolia* butanol extract. (A) negative and (**B**) positive ion modes..

**Fig 3:**
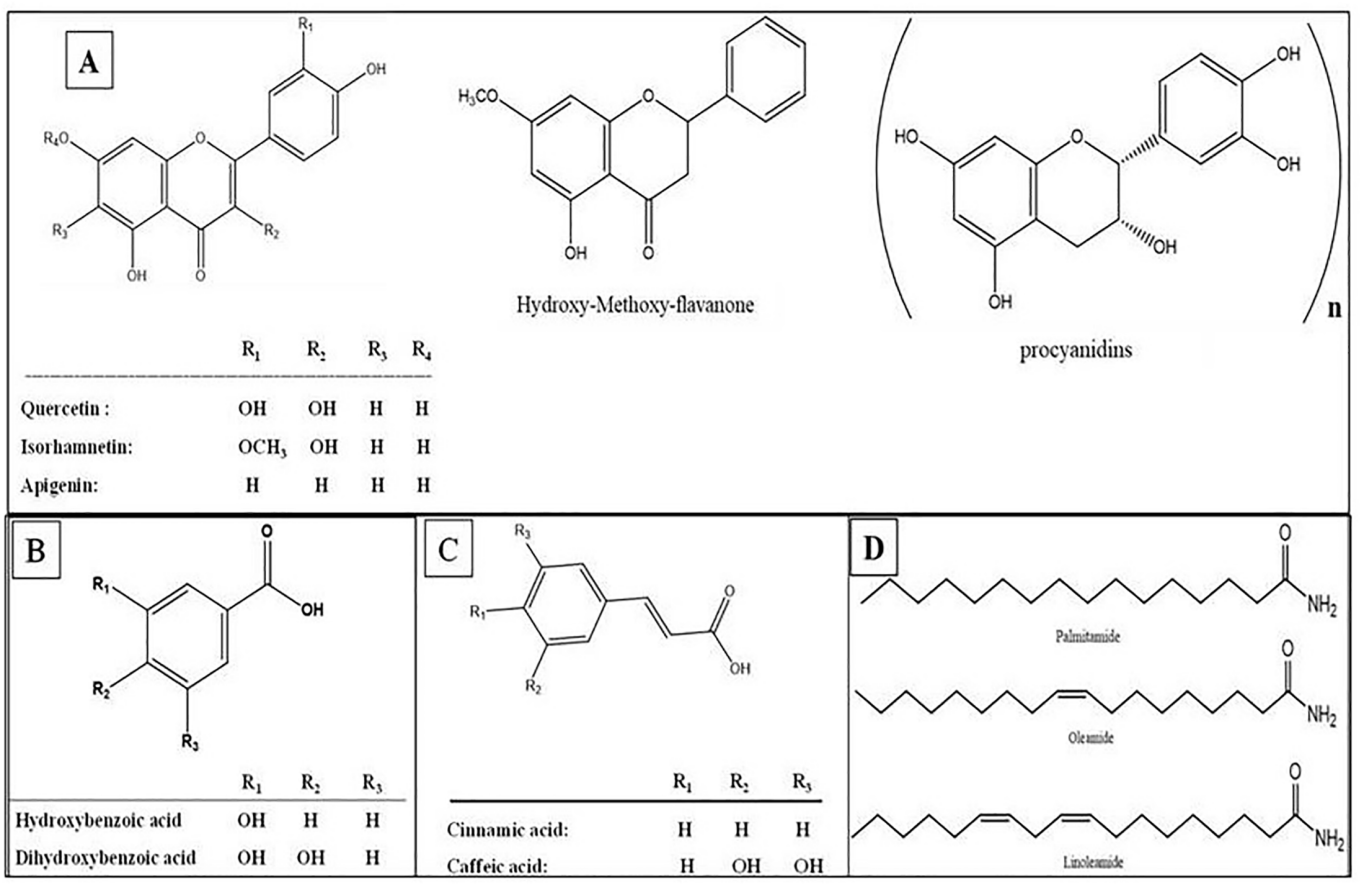
Chemical structures of the main identified metabolic classes identified from of *G. ulmifolia* butanol extract.

Reversed-phase UPLC/PDA/ESI-qTOF-MS analysis was employed to profile metabolite of *G. ulmifolia* butanol extract (Gul-BuOH) in an untargeted approach. To the best of our knowledge, this represents the first report for profiling *G. ulmifolia* phytocomponents leading to the annotation of a total of (42) metabolites belonging to different classes, including flavonoids (13), phenolic acids (8), organic acids (6), alcohols (2), fatty acids (3), sugars (2), and fatty acid amides (8) (Table 2). UPLC-PDA-ESI–qTOF-MS/MS negative and positive ion modes chromatogram of Gul-BuOH and chemical structures of the main identified phytochemical classes are shown in Figs 2 and 3, respectively. Metabolites were eluted based on polarity, starting with the most polar compounds, i.e., sugar, organic acids, phenolic acids, and alcohols, followed by compounds of moderate polarity, i.e., flavonoids, and the least polar metabolites, i.e., fatty acids and fatty acid amides, which were eluted last in the chromatogram. The analysis of Gul-BuOH was conducted using both ionization modes of electrospray ionization (ESI) to achieve a comprehensive metabolome profile. The negative ionization mode demonstrated superior sensitivity, producing pronounced [M-H]^-^ ions and achieving higher signal-to-noise ratios with less background noise compared to the positive ionization mode.

**Table 2:** Phytochemical components annotated in *G. ulmifolia* butanol extract using UPLC-PDA-ESI–qTOF-MS/MS in negative/ positive ionization modes.

No.	Rt. (min)	UV	Identification	Class	[M-H]^-^/[M + H]^+^ (m/z)	Error (ppm)	Elemental composition	MS^n^ product ions
1	0.45	ND	Disaccharide	Sugar	341.10892	−0.043	C_12_H_21_O_11_^-^	179, 143,
2	0.48	ND	Caffeoyl-*O*-hexoside derivative	Phenolic acid	377.086	−4.788	C_18_H_17_O_9_^-^	341, 215, 179
3	0.66	252	Malic acid	Organic acid	133.0146	2.657	C_4_H_5_O_5_^-^	115, 87, 71
4	0.76	ND	Succinic acid	Organic acid	117.01964	2.632	C_4_H_5_O_4_^-^	99, 73
5	0.87	ND	Disaccharide isomer	Sugar	341.10876	−0.512	C_12_H_21_O_11_^-^	179, 161, 143, 113
6	1.04	279	Dihydroxybenzoic acid-*O*-hexoside	Phenolic acid	315.07211	−0.144	C_13_H_15_O_9_^-^	165, 153, 151, 109
7	1.56	258, 290	Protocatechuic acid (Dihydroxybenzoic acid)	Phenolic acid	153.01955	1.425	C_7_H_5_O_4_^-^	109, 135, 123
8	1.77	279	Caffeic acid-rhamnosyl derivative (Decaffeoylacteoside/Decaffeoylverbascoside)	Phenolic acid	461.16611	−0.736	C_20_H_29_O_12_^-^	315, 297, 179, 161, 135,
9	2.51	281	Caffeic acid-pentosyl derivative	Phenolic acid	447.15063	−0.379	C_19_H_27_O_12_^-^	401,343, 315, 191, 179, 149, 135
10	2.64	290	Unknown	Unknown	349.05966	−7.788	C_9_H_17_O_14_^-^	331, 241, 181, 165, 151, 139
11	2.78	290	Caffeic acid hexoside	Phenolic acid	341.08777	−0.104	C_15_H_17_O_9_^-^	179, 135
12	2.92	286	Dihydroxybenzoic acid-*O*-hexosyl-pentoside	Phenolic acid	447.18683	−0.794	C_18_H_23_O_13_^-^	401, 343, 315, 239, 221, 179, 153
13	4.41	290	Caffeic acid-pentosyl derivative	Phenolic acid	431.15558	−0.305	C_19_H_27_O_11_^-^	299, 233, 191, 179, 161, 149, 131, 113
14	7.58	369	Unknown	Unknown	523.16644	−0.403	C_21_H_31_O_15_^-^	477, 293
15	7.72	369	Benzyl alcohol pentosyl-hexoside	phenolic alcohol	401.14499	−0.33	C_18_H_25_O_10_^-^	383, 293, 269, 233, 191, 161
16	7.91	286, 369	Proanthocyanidin	Flavonoid	577.13446	−0.689	C_30_H_25_O_12_^-^	451, 433, 425, 407, 299, 289, 287, 245
17	8	213, 343	Methyl jasmonate-*O*-hexoside	Organic acid	385.18665	−0.36	C_19_H_29_O_8_^-^	387, 329, 315, 299, 191, 179, 161, 149, 143, 131, 125, 113
**18**	8.16	213, 343	benzyl alcohol pentosyl-hexoside isomer	phenolic alcohol	401.14511	−0.21	C_18_H_25_O_10_^-^	383, 293, 269, 191, 161
**19**	8.36	213, 341	Methyl jasmonate-*O*-hexoside isomer	Organic acid	385.18665	−0.366	C_19_H_29_O_8_^-^	223, 205, 179, 161, 153
**20**	8.64	215, 290, 334	Unknown	Unknown	445.13474	−0.409	C_19_H_25_O_12_^-^	413, 399, 341, 313, 293, 281, 191, 161,
21	8.73	215, 290, 334	Methyl jasmonate-*O*-pentosyl-hexoside	Organic acid	517.22845	−1.159	C_24_H_37_O_12_^-^	447, 431, 401, 293
22	8.86	216, 286, 349	Unknown	Unknown	641.20813	−0.05	C_29_H_37_O_16_^-^	623, 611, 597, 530, 519, 477, 461, 323, 239
23	8.97	216, 286, 338	Vicenin 2	Flavonoid	593.15094	−0.253	C_27_H_29_O_15_^-^	575, 533, 515, 503, 473, 455, 413, 383, 353, 285
24	9.05	282, 334	Proanthocyanidin trimer	Flavonoid	865.19739	−0.69	C_45_H_37_O_18_^-^	847, 739, 713, 695, 677, 587, 577, 575, 543, 533, 451, 425, 407, 289, 287
25	9.14	283, 334	Unknown	Unknown	249.08028	3.433	C_13_H_13_O_5_^-^	97
26	9.19	217, 283, 337	Unknown	Unknown	641.20831	0.23	C_29_H_37_O_16_^-^	623, 611, 597, 531, 517, 495, 461, 443, 315, 197
27	9.3	282, 334	Unknown	Unknown	415.16086	−0.11	C_19_H_27_O_10_^-^	397, 293, 233, 191, 179, 161, 149, 143, 131, 113
28	9.33	282, 337	Apigenin-6-*C*-hexoside-8-*C*-pentoside	Flavonoid	563.14026	−0.655	C_26_H_27_O_14_^-^	545, 517, 503, 473, 443, 425, 413, 383, 353
29	9.41	281, 335	Methyl jasmonate-*O*-hexoside isomer	Organic acid	385.18668	−0.288	C_19_H_29_O_8_^-^	225, 205, 179, 161, 143
30	9.49	282, 334	Unknown	Unknown	514.28424	−5.72	C_37_H_38_O_2_^-^	435, 425
31	9.52	282, 334	Unknown	Unknown	581.22375	−0.369	C_28_H_37_O_13_^-^	566, 535, 427, 419, 409, 401, 386, 373, 337, 233
32	9.56	282, 333	Isorhamentin-*O*-rhamnosyl-di-hexoside	Flavonoid	785.25031	−0.836	C_35_H_45_O_20_^-^	704, 623, 461, 315
33	9.71	282, 334	Quercetin-*O*-rhamnosyl-hexoside	Flavonoid	609.14575	0.31	C_27_H_29_O_16_^-^	447, 343, 301, 299, 271, 255, 179
34	9.83	282, 334	Isorhamentin-*O*-hexosyl-rhamonside	Flavonoid	623.19812	−0.038	C_28_H_31_O_16_^-^	477, 461, 443, 315, 179
35	10.22	284, 330	Di-hydroxy-di-methoxy-flavanone-*O*-di-rhamnoside	Flavonoid	607.20313	−0.163	C_29_H_35_O_14_^-^	461, 315, 299, 179
36	10.29	283, 324	Isorhamentin-*O*-hexosyl-rhamonside isomer	Flavonoid	623.19812	−0.08	C_28_H_31_O_16_^-^	461, 315, 179
37	10.34	283, 328	Di-hydroxy-di-methoxy-flavanone-*O*-di-rhamnoside isomer	Flavonoid	607.203	−0.377	C_29_H_35_O_14_^-^	461, 315, 299, 179
38	10.5	283, 324	Hydroxy-di-methoxy-flavanone-*O*-di-rhamnoside	Flavonoid	591.20795	−0.616	C_29_H_35_O_13_^-^	545, 471, 453, 445, 427, 299
39	10.73	282, 324	Hydroxy-methoxy-flavanone-*O*-rhamnosyl-hexoside	Flavonoid	577.19232	−0.596	C_28_H_33_O_13_^-^	559, 531, 431, 415, 355, 337, 269, 193, 179
40	11.05	283, 334	Hydroxy-di-methoxy-flavanone-*O*-di-rhamnoside isomer	Flavonoid	591.20776	−0.937	C_29_H_35_O_13_^-^	547, 445, 425, 427, 385, 325, 295, 265, 179
41	11.09	286, 334	Unknown	Unknown	593.12976	−0.512	C_30_H_25_O_13_^-^	447,307, 285, 257
42	11.7	ND	Trihydroxy-octadecadienoic acid	Fatty acid	327.21759	−0.328	C_18_H_31_O_5_^-^	309, 291, 273, 229, 211, 171
43	12.03	ND	Trihydroxy-octadecenoic acid	Fatty acid	329.23337	0.069	C_18_H_33_O_5_^-^	314, 311, 309, 293, 229, 211, 171
44	13.12	ND	Dihydroxy-methyl-hexadecatetraenoic acid	Fatty acid	293.17578	−0.179	C_17_H_25_O_4_^-^	236, 221, 193
45	14.27	224	Unknown	Unknown	311.16858	−8.23	C_13_H_27_O_8_^-^	293, 247, 197, 183
46	16.13	ND	Linoleamide	Fatty acyl amide	280.264	1.637	C_18_H_34_NO^+^	263, 245,
47	16.45	ND	Palmitamide	Fatty acyl amide	256.264	2.259	C_16_H_34_NO^+^	228, 214, 200, 186, 172, 158, 144, 130, 116
48	16.68	ND	Oleamide	Fatty acyl amide	282.2795	1.731	C_18_H_36_NO^+^	265, 247
49	17.55	ND	Stearamide	Fatty acyl amide	284.2953	1.86	C_18_H_38_NO^+^	series loss of CH_2_
50	17.72	ND	Eicosenamide	Fatty acyl amide	310.3112	2.412	C_20_H_40_NO^+^	293, 275 -series loss of CH_2_
51	18.64	ND	Eicosanamide	Fatty acyl amide	312.3266	1.628	C_20_H_42_NO^+^	295, 286 -series loss of CH_2_
52	18.73	ND	Docos-13-enamide	Fatty acyl amide	338.3424	1.798	C_22_H_44_NO^+^	321, 303, 296 -series loss of CH_2_
53	19.89	ND	Docosanamide	Fatty acyl amide	340.3579	1.435	C_22_H_46_NO^+^	322, -series loss of CH_2_

ND: Not detected.

#### 3.1.1 Identification of flavonoids.

Thirteen chromatographic peaks (**16**, **23**, **24, 28, 32–40**) were annotated as flavonoids and their conjugates. The identified flavonoids belonged to flavonol **(32–34** & **36**), flavan-3-ol **(16** & **24)**, flavanone (**35, 37–40**), and flavone (**23** & **28**) as shown in Table 2, with a notable abundance of methoxylated flavonoids, represented by 8 peaks. Methoxylated flavonoids are less common in nature than other flavonoid conjugates, with the additional advantage of being more biologically active due to their increased lipophilicity [40]. Biological activities attributed to methoxylated flavonoids include antioxidant effect, antiproliferative activity, Na^+^, K^+^-ATPase inhibition, and Adenosine A1 binding affinity leading to a diuretic effect [41]. Tandem mass analysis was further utilized for the assignment of aglycone in flavonoid glycoside type *O*- versus *C*-glycosides. *O*-Glycosides showed neutral loss of −132, −146, and −162 amu corresponding to pentose, deoxyhexose, and hexose, respectively [42]. In contrast, *C*- type glycosides showed the loss of −60, −90, and −104 amu for pentoses, −74, −104 and −134 amu for deoxyhexoses, and −90, −120, and −134 amu for hexoses, indicative of cross-ring cleavages (0,3, 0,2, and 1,5, respectively) of the sugar moiety [43].

#### 3.1.2 Flavonols.

Four flavonol glycosides (**32**, **33**, **34** & **36**), were identified as the *O*-glycoside type. Isorhamnetin conjugates were the most abundant flavonols in Gul-BuOH appearing in three peaks, for example peak **32** with molecular mass of 785.25031 and a predicted formula C_35_H_45_O_20_^–^, showed losses of two −162, −146 amu corresponding to two hexosyl and rhamnosyl moieties, respectively, yielding aglycone fragment ion at *m/z* 315 for isorhamnetin, annotated as isorhamnetin-*O*-rhamnosyl-di-hexoside. Similarly, peak **33,** M-H^-^ at *m/z* 609.14575, C_27_H_29_O_16_^–^, showed a fragmentation pattern of *O*-glycosides with respective losses −162 and −146 amu yielding aglycone ion at *m/z* 301 for quercetin, and annotated as quercetin-*O*-rhamnosyl-hexoside (S1 Fig). These flavonols have not previously been identified in *G. ulmifolia*, offering new insights into its chemical profile. Flavonol glycosides like isorhamnetin and quercetin previously exhibited hepatoprotective effects, with isorhamnetin reducing liver lipid accumulation and fibrosis. It works by inhibiting the de novo lipogenesis (DNL) pathway and downregulating key lipogenic and fibrotic genes like sterol regulatory element-binding protein 1c (SREBP1c), fatty acid synthase (FAS), acetyl-Coenzyme A carboxylase alpha (ACC1), transforming growth factor beta (TGFβ), and collagen. [44]

#### 3.1.3 Flavan-3-ols.

The (-)ESI-MS/MS spectra of dimeric procyanidin (Peak **16**) at *m/z* 577.1344, yielded [M-H-152]^-^ fragment ions at *m/z* 425 from Retro-Diels-Alder (RDA) rearrangement of the heterocyclic ring, at *m/z* 407 ([M-H-170]) from RDA-F of the heterocyclic ring and loss of H_2_O, at *m/z* 451 ([M-H-126]^-^) from cleavages between C_4_-C_5_ and O-C_2_ of one pyran ring and at *m/z* 289 ([M-H-289]^-^) from cleavage of the interflavanic bond, respectively (S2 Fig) [45]. Similarly, peak **24** was annotated as proanthocyanidin trimer, from its molecular mass at *m/z* 865.19739 and formula of C_45_H_37_O_18_^–^. Both procyanidin dimer and trimer were previously reported in *G. ulmifolia* fruits [14]. Both have potential hepatoprotective effects due to their strong antioxidant and anti-inflammatory properties. Procyanidin timer, in particular, reduces oxidative stress by scavenging free radicals, decreasing lipid peroxidation, and modulating inflammatory pathways like NF-κB and MAPK signaling, which protect liver cells from damage and apoptosis, offering significant protection against liver toxicity, especially in cases induced by heavy metals like cadmium [46].

#### 3.1.4 Flavanones.

Flavanones were annotated in 5 peaks **35, 37–40**. The identified flavanone conjugates yielded a fragmentation pattern typical of *O*-type glycosides. For example, peak **35** with M-H^-^ at *m/z* 607.2031, C_29_H_35_O_14_^–^ showed respective losses of two −146 amu corresponding to two rhamnosyl moieties, to yield aglycone fragment at *m/z* 315 corresponding to di-hydroxy-di-methoxy-flavanone, annotated as di-hydroxy-di-methoxy-flavanone-*O*-di-rhamnoside. Peak **39** was annotated as hydroxy-methoxy-flavanone-rhamnosyl-hexoside, from its M-H^-^ at *m/z* 577.19232, C_28_H_33_O_13_^–^, with respective losses of −162 and −146 amu equivalent to hexosyl and rhamnosyl moieties. To the best of our knowledge, this is the first report of these flavanones in *G. ulmifolia*.

#### 3.1.5 Flavones.

Two apigenin flavone glycosides of *C*-type were detected in *G. ulmifolia* (peaks **23** & **28**). For example, peak **23,** M-H^-^ at *m/z* 593.1509, C_27_H_29_O_15_^-^ was classified as *C*-glycoside type from its fragmentation pattern (S3 Fig), showing the losses of −90 and −120 amu appearing at *m/z* 503 and 473, followed by further losses of −90 and −120 amu appearing at *m/z* 383 and 353, indicative for the presence of two hexose units and was annotated as vicenin 2 (apigenin-6,8-di-*C*-hexoside). Similarly, peak **28** at *m/z* 563.1402, C_26_H_27_O_14_^-^ was annotated as apigenin-6-*C*-hexoside-8-*C*-pentoside based on fragment intensities as previously mentioned [47]. The presence of flavone C-glycosides in *G. ulmifolia* offers new insights into its potential therapeutic effects, particularly in liver diseases. Furthermore, this is the first report of flavone *C*-glycosides in *G. ulmifolia*, contributing to its emerging role in liver protection [48]

#### 3.1.6 Phenolic acids.

Phenolic acids are commonly identified as precursors for several phenolic metabolites, detected either as free or conjugated with sugars and various organic acids. Owing to their significant polarity, phenolic acids were detected in negative ion mode, due to their acidic nature and showing [M − H-44]^−^ fragment ion corresponding to decarboxylation [49]. Eight phenolic acids were identified in Gul-BuOH, five of which (**2**, **8**, **9, 11 & 13**) belonged to hydroxycinnamic acid derivatives, whereas the remaining three peaks (**6**, **7** & **12**) belonged to hydroxybenzoates, as shown in Table 1.

The identified cinnamates were detected as conjugation with sugars, with caffeic acid as the main aglycone. The predominant fragment at *m/z* 179 equivalent to caffeic acid in the MS^n^ spectrum in peak **11** (*m/z* 341.0877, C_15_H_17_O_9_^-^) with extra loss of −162 amu due to a hexose moiety was annotated as caffeic acid-*O*-hexoside (S4 Fig). Caffeic acid conjugate was likewise detected in peaks **2, 9, and 13** as shown in Table 1 [50]. Compared to cinnamates, derivatives of hydroxybenzoic acid displayed a distinctive fragment at *m/z* 153 and 135 corresponding to dihydroxy and hydroxybenzoic acids, respectively [47]. For example, peak **6** at *m/z* 315.07211, C_13_H_15_O_9_^-^ with predominant fragment of 153 amu for dihydroxybenzoic acid with extra loss of −162 amu, mass spectrum showed characteristic fragments at *m/z* 135, and 109 due to loss of [M-162-H_2_O]^-^, and [M-162-CO_2_]^-^, respectively, so it was annotated as dihydroxybenzoic acid-*O*-hexoside **(**S5 Fig**)**. The phenolic acids in *G. ulmifolia*, particularly caffeic acid and its derivatives, exhibit antioxidative and anti-inflammatory effects, which are crucial in mitigating liver damage and steatosis [51]. This profile is consistent with previously published data on *G. ulmifolia* fruits, reinforcing the potential of phenolic acids in managing metabolic dysfunction-associated steatotic liver disease [52].

#### 3.1.7 Organic acids.

Four organic acids (**3**,**4**, **17**, **19**, **21** & **29**) were detected early in the chromatogram owing to their high polarity, identified based on MS^2^ fragments of −18 and −44 amu due to loss of H_2_O molecules and carboxyl group, respectively [53]. Major identified acids included malic acid and succinic acid in peaks **3** and **4** (S6 Fig), with this being the first report of these organic acids in *G. ulmifolia*.

#### 3.1.8 Fatty acids.

In the last part of the chromatographic run, three fatty acids (**42–44**) were eluted due to their nonpolar nature. The identified unsaturated fatty acids were oxygenated, *i.e.*, dihydroxy (**44**) and trihydroxy (**42** & **43**). For example, peak **42** annotated as trihydroxy octadecadienoic acid, showed M-H^-^ at *m/z* 327.2175 C_18_H_31_O_5_^-^ and three characteristic fragments at *m/z* 309, 291, and 273 due to three successive losses of H_2_O, alongside another key fragment appearing at *m/z* 283 due to the loss of CO_2_. Hydroxy fatty acids have been previously suggested to play a role in mediating anti-inflammatory and anticancer activities [47].

#### 3.1.9 Fatty acyl amides.

Generally, FAAs are bioactive lipid signaling molecules that play key roles in several biological activities, such as to inhibit the migration of cancer cells and anti-inflammatory effect which may be play a role in hepatoprotective activity, albeit, the overall role of fatty acyl amides is still under investigation, [54] and are the first time to be reported in *G. ulmifolia.* In this study, a total of eight peaks (**46−53**) were observed as the [M + H]^+^ ion in positive ion mode (ESI+), considering improved detection of nitrogen in that ion mode. Out of these, four peaks were annotated as saturated fatty acyl amides showing similar fragmentation, regardless of the acyl chain length, ranging from C_16_ to C_22_. These peaks were annotated as palmitamide (**47**, S7 Fig), stearamide (**49**), eicosanamide (**51**), and docosanamide (**53**). The MS/MS spectra of these peaks showed fragments corresponding to the cleavage of the acyl fragmentation site as observed in oleamide [C18:1], eicosenamide [C20:1], and docos-13-enamide [C22:1]. In addition to saturated and monounsaturated fatty acyl amides, di-saturated fatty acid amide was detected in peak **46**, annotated as linoleamide [C18:2].

### 3.2 Biological results

#### 3.2.1. Effects of Gul-BuOH on Cd accumulation in liver tissue.

**Fig 4:**
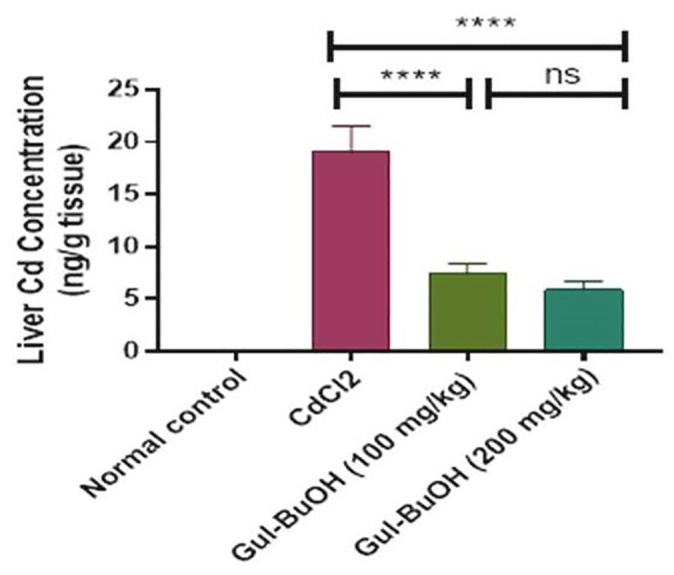
Ameliorative effects of *G. ulmifolia* butanol extract on liver Cd concentration in CdCl_2_-induced liver toxicity in rats. Each bar represents the mean ± SEM. (n = 6), ^ns^ when P > 0.05, ^*^ when P ≤ 0.05 ^**^ when P ≤ 0.01, ^***^ when P ≤ 0.001 and ^****^ when P ≤ 0.0001.

Exposure of rats to CdCl_2_ led to a marked increase in hepatic Cd accumulation compared with the untreated normal control group. In contrast, concomitant administration of Gul-BuOH (100 and 200 mg/kg) significantly reduced Cd levels in liver tissues at both dose levels by 2.6 and 3.2 folds, respectively relative to the CdCl₂-intoxicated group (Fig 4).

#### 3.2.2 Effects of Gul-BuOH on oxidative stress and antioxidant parameters.

**Fig 5:**
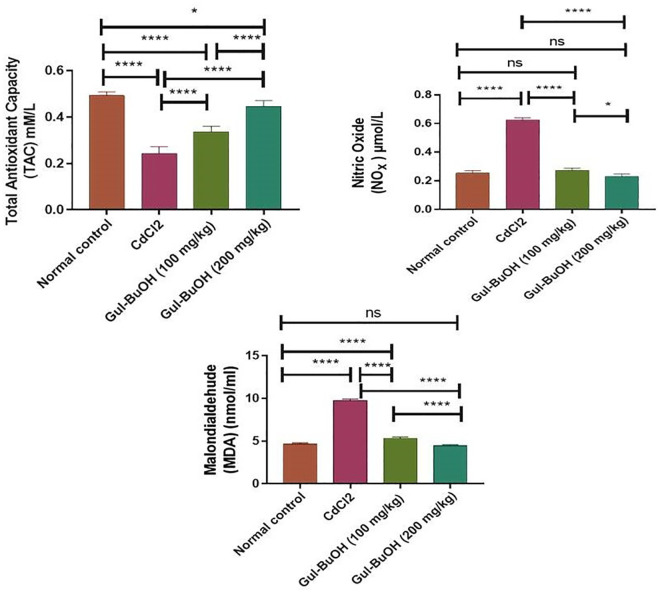
Effect of *G. ulmifolia* butanol extract on antioxidant parameters (TAC, NO and MDA) in CdCl_2_-induced liver toxicity in rats. Each bar represents the mean ± SEM. (n = 6), ^ns^ when P > 0.05, ^*^ when P ≤ 0.05 ^**^ when P ≤ 0.01, ^***^ when P ≤ 0.001 and ^****^ when P ≤ 0.0001.

As shown in Fig 5, CdCl_2_ exposure markedly impaired hepatic antioxidant defenses, evidenced by a 2.0-fold reduction in TAC, along with significant elevations in NO and MDA levels by 2.5- and 2.1-fold, respectively, relative to normal controls. Administration of Gul-BuOH (100 and 200 mg/kg) effectively counteracted these alterations, restoring TAC by 38.3% and 83.2%, and reducing NO by 56.3% and 63.4% as well as MDA by 45.4% and 54.6%, respectively.

#### 3.2.3. Effects of Gul-BuOH on liver function enzymes.

**Fig 6:**
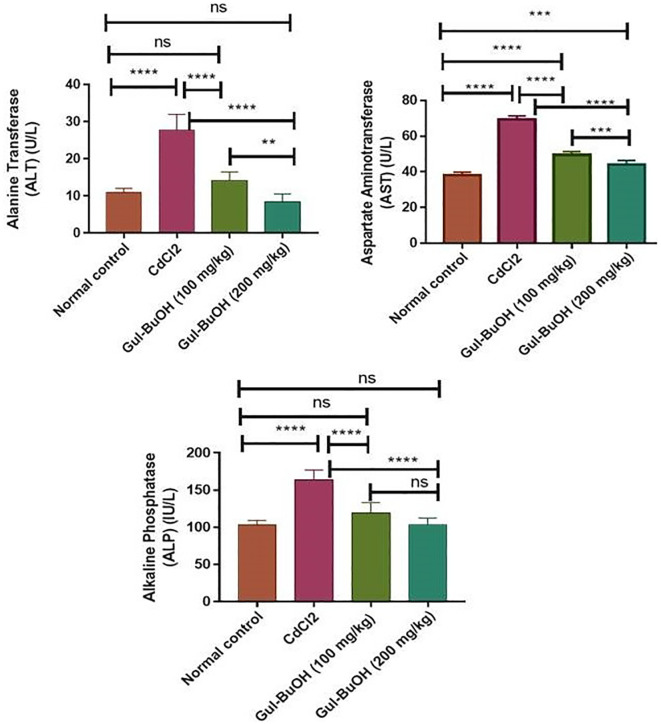
Effect of *G. ulmifolia* butanol extract on liver function enzymes (ALT, AST and ALP) in CdCl_2_-induced liver toxicity in rats. Each bar represents the mean ± SEM. (n = 6), ^ns^ when P > 0.05, ^*^ when P ≤ 0.05 ^**^ when P ≤ 0.01, ^***^ when P ≤ 0.001 and ^****^ when P ≤ 0.0001.

The CdCl_2_ administration significantly disrupted hepatic function, as indicated by elevations in serum ALT, AST, and ALP activities by 2.6-, 1.8-, and 1.6-fold, respectively, compared with normal controls. Treatment with Gul-BuOH (100 and 200 mg/kg) markedly ameliorated these disturbances, reducing ALT levels by 48.8% and 69.9%, AST by 28.8% and 36.7%, and ALP by 27.2% and 36.7%, respectively (Fig 6).

#### 3.2.4 Effects of Gul-BuOH on anti-inflammatory markers.

**Fig 7:**
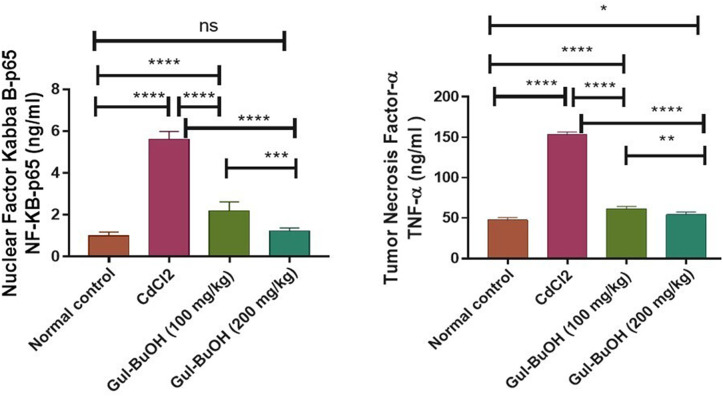
Effect of *G. ulmifolia* butanol extract on anti-inflammatory markers (NF-κB-p and TNF-α) in CdCl_2_-induced liver toxicity in rats. Each bar represents the mean ± SEM. (n = 6), ^ns^ when P > 0.05, ^*^ when P ≤ 0.05 ^**^ when P ≤ 0.01, ^***^ when P ≤ 0.001 and ^****^ when P ≤ 0.0001.

As illustrated in Fig 7, CdCl_2_ exposure markedly up regulated inflammatory mediators, with serum NF-κB-p and TNF-α levels elevated by 5.7- and 3.2-fold, respectively, relative to normal controls. Administration of Gul-BuOH (100 and 200 mg/kg) substantially attenuated these increases, lowering NF-κB-p by 61.1% and 78.2% and TNF-α by 59.8% and 64.6%, respectively.

#### 3.2.5 Effects of Gul-BuOH on quantification of liver mi RNA Let7a and Inc RNA HOTAIR gene expression in liver tissue homogenate.

**Fig 8:**
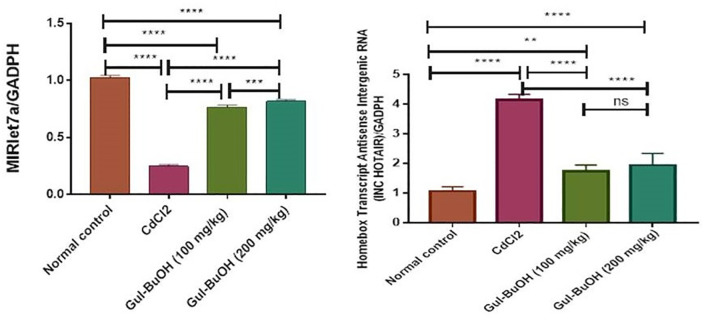
Effect of *G. ulmifolia* butanol extract on quantification of liver mi RNA Let7a and Inc RNA HOTAIR gene expression in CdCl_2_-induced liver toxicity in rats. Each bar represents the mean ± SEM. (n = 6), ^ns^ when P > 0.05, ^*^ when P ≤ 0.05 ^**^ when P ≤ 0.01, ^***^ when P ≤ 0.001 and ^****^ when P ≤ 0.0001.

The CdCl_2_ exposure markedly down regulated mi RNA Let7a expression by 4.2, and increase Inc RNA HOTAIR expression by 3.9-fold, relative to normal controls. Administration of Gul-BuOH (100 and 200 mg/kg) substantially attenuated down regulation of mi RNA Let7a by 212.2% and 232.7% and decrease Inc RNA HOTAIR by 58.2% and 53% (Fig 8).

#### 3.2.6 Effects of Gul-BuOH on the CdCl_2_‑induced histopathological alterations in liver tissues.

**Fig 9:**
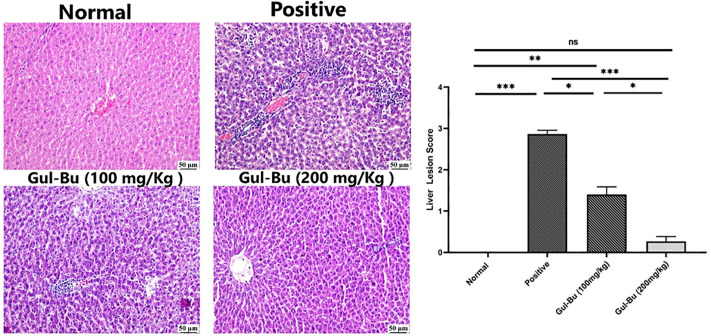
The histopathological findings of liver from different experimental groups (H&E). Normal hepatocytes arranged in hepatic cords around normal central vein (cv) of control negative group, Cadmium treated group showing necrobiotic changes of hepatocytes with multifocal aggregation of mononuclear inflammatory cells and congestion of central vein and, Gul-BuOH (100 mg/kg) exhibit multifocal mononuclear cells aggregates hepatocellular degeneration and necrosis. Normal hepatocytes of high dose group of Gul-BuOH (200 mg/kg) with normal portal area. Chart present the liver lesion score, the values are expressed as the mean ± standard error of mean (SEM) using Kruskal-Walli’s test followed by Dunn’s Multiple Comparison test. ^ns^ when P > 0.05, ^*^ when P ≤ 0.05 ^**^ when P ≤ 0.01 and ^***^ when P ≤ 0.001.

As demonstrated in Fig 9, normal histology of hepatocytes and portal triad was found in the liver tissue of control negative group. Regarding the histopathological finding of the CdCl_2_-induced group multifocal inflammatory cells infiltration in various lobules with necrobiotic changes of hepatocytes was present that occupied almost of hepatic lobules in addition to congestion of some central veins and hepatic sinusoids. Group 3 treated with Gul-BuOH (100 mg /kg), hepatic sections exhibited hepatocellular degeneration and necrosis, sinusoidal congestion, multifocal aggregation of mononuclear inflammatory cells. Marked improvement was detected in high dose of Gul-BuOH (200 mg/kg) that indicated with apparently normal portal triad structure and hepatocytes in approximately of the examined hepatic lobules. The hepatic injury subsided with high doses of Gul-BuOH that confirmed with histopathological alterations. A higher lesion score was observed in Cadmium treated group compared to other experimental groups, there was a significance difference between both Gul-BuOH groups, and moreover no statistically significant difference was detected between high dose group of Gul-BuOH and normal groups.

#### 3.2.7 Immunohistochemical analysis of HO-1 and Sirt-1 in liver tissue.

**Fig 10:**
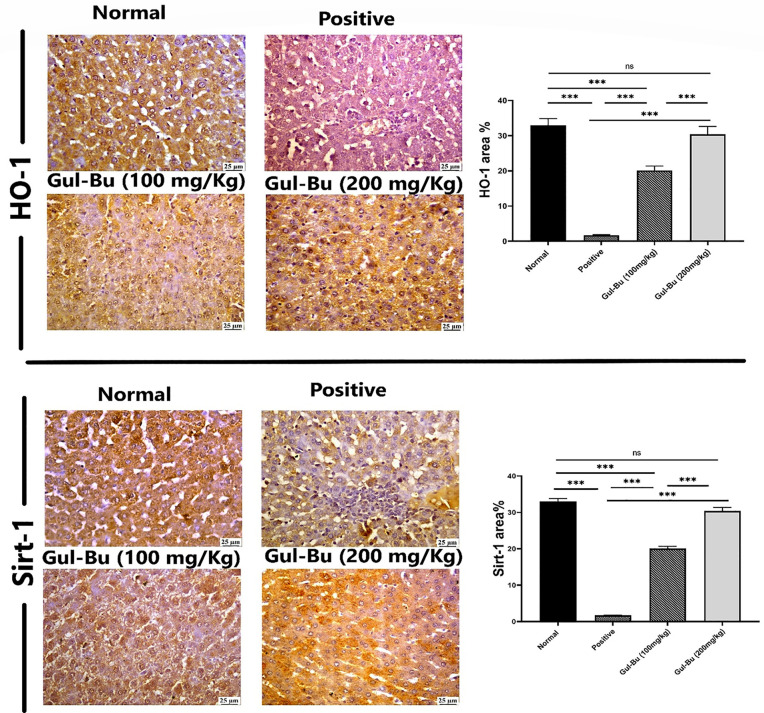
Immunoreactivity of HO-1 and Sirt-1 proteins in hepatocytes of different experimental groups. Upper panel presenting the immunoreactivity of HO-1 expression in hepatic section, Gul-BuOH improved CdCl_2_-induced impairment in hepatic antioxidant in rats. Contrary to the CdCl_2_ group downregulated the HO-1 expression in hepatic section. Chart present HO-1 area %. Lower panel presenting the immunoreactivity of Sirt-1 expression in hepatic section, Gul-BuOH upregulated Sirt-1 expression in CdCl_2_-induced impairment. Contrary to the CdCl_2_ group downregulated the Sirt-1 expression in hepatic sections. Chart present Sirt-1 area %, the values are expressed as the mean ± standard error of the mean (SEM) using one-way factorial analysis of variance (ANOVA) ^ns^ when P > 0.05, ^*^ when P ≤ 0.05 ^**^ when P ≤ 0.01 and ^***^ when P ≤ 0.001.

Intense immunoreactivity against HO-1 was detected in the hepatic sections of normal group. In contrast to the CdCl_2_-treated group that showed minimal reactivity against HO-1 in the cytoplasm of hepatocytes. The immunoreactivity was moderate in Gul-BuOH (100 mg/Kg) group that gradually increased high dose groups respectively. Evaluation of HO-1 area % revealed a significance difference between experimental groups as the expression was upregulated in all treated groups in compared to CdCl_2_ treated group. Interestingly, there was no statistical significance difference between high dose of Gul-BuOH and normal groups (Fig 10). Sirt-1 was stimulated with in groups treated with Gul-BuOH against the CdCl_2_ induced impairment as detected in the examined hepatic section. The immunoreactivity was moderate in GU-BuOH (100 mg/Kg) group that gradually increased high dose groups respectively. Evaluation of Sirt-1 area % revealed a significance difference between experimental groups as the expression was upregulated in all treated groups in compared to CdCl_2_ treated group. Interestingly, there was no statistical significance difference between high dose of Gul-BuOH and normal groups.

#### 3.2.8 Serum metabolomics for revealing metabolic alterations between CdCl_2_-induced hepatotoxicity and Gul-BuOH-treated rats.

**Fig 11:**
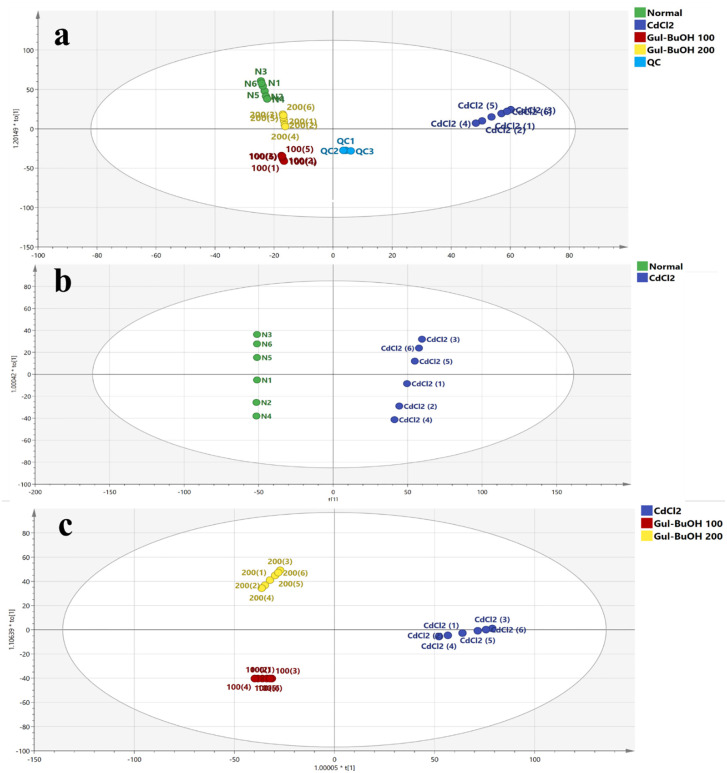
Score scatter plot of OPLS-DA models. (**A**) all five groups (normal, cadmium chloride-induced hepatotoxicity, quality control and two dose levels-based Gul-BuOH- treated groups), (**B**) normal and CdCl_2_-induced hepatotoxicity groups and (C) CdCl_2_-induced hepatotoxicity and dose-based Gul-BuOH-treated groups.

To further elucidate the metabolic mechanisms underlying the hepatoprotective activity of Gul-BuOH, untargeted serum metabolomics profiling by GC–MS coupled with chemometric analysis was employed for the first time to explore its therapeutic impact. The supervised OPLS-DA model generated from the GC–MS dataset (Fig 11) revealed a clear distinction between the diseased group and both the healthy controls and treated groups. Model performance indicators demonstrated strong reliability, with an R^2^Y (explained variance) of 0.991 and a Q^2^ (predictive variance) of 0.988. The clustering of pooled quality control (QC) samples near the model center confirmed that the orthogonal components effectively captured systematic variation, allowing the predictive component to isolate biologically meaningful differences among the groups (Fig 11A). The tight QC clustering also attests to the high analytical stability of the method.

The validity of the multivariate model was assessed using CV-ANOVA implemented in SIMCA software. The obtained CV-ANOVA results demonstrated a highly significant model performance with an F-value of 267.637 and an extremely low p-value (P < 0.001). These findings indicate that the predictive variation explained by the model is significantly greater than the residual variation, confirming the robustness, reliability, and non-random nature of the model with minimal risk of overfitting. The low residual sum of squares (0.867) compared with the regression sum of squares (103.133) further supports the excellent goodness-of-fit and predictive capability of the model. Permutation testing (20 permutations) was performed to evaluate the robustness and predictive validity of the OPLS-DA model and to exclude model overfitting. In all models, the original model displayed substantially higher R^2^ and *Q*^2^ values than those obtained from the permuted models. Moreover, the *Q*^2^ regression line showed a negative or near-zero intercept, indicating that the observed class discrimination was not generated by random chance. These findings confirm the stability, reliability, and predictive capability of the constructed OPLS-DA model (S9 Fig**)**

In the OPLS-DA scores plot, the CdCl_2_-induced hepatotoxicity group appeared on the positive side of the first latent variable (LV1), whereas the healthy group and all Gul-BuOH-treated groups at the two dose levels were positioned on the negative side (Fig 11A). The second latent variable (LV2) further differentiated subclasses: the lower-left quadrant contained the low-dose of Gul-BuOH-treated group (100 mg/kg), while the upper-left quadrant contained both the high-dose group (200 mg/kg) and the normal controls (Fig 11A). This separation pattern strongly suggests that treatment with Gul-BuOH at 200 mg/kg restores the serum metabolic profile of the hepatotoxicity-induced group toward that of the healthy state. Moreover, the observed group segregation highlights distinct metabolic signatures in the liver toxicity model that differ markedly from those of **the normal rats** (Fig 11B) as well as from all two Gul-BuOH treatment groups (Fig 11C).

**Fig 12:**
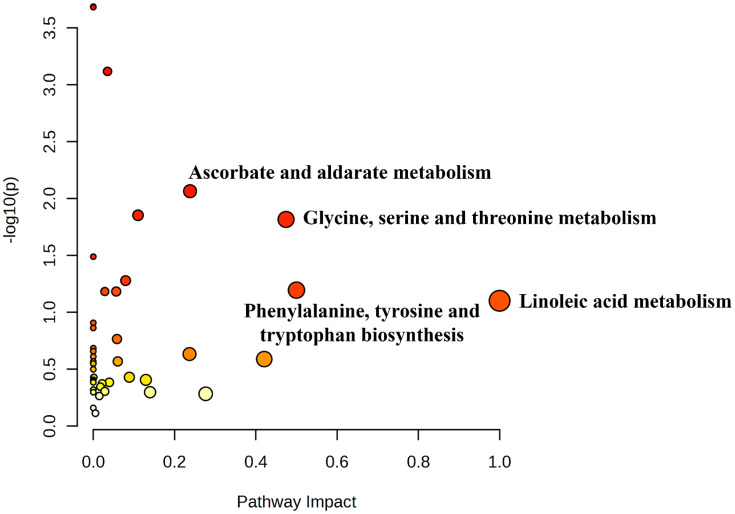
Key metabolic pathways revealed by enrichment analysis of differential metabolites in rats’ sera. The node size indicates the pathway impact value.

Univariate statistical analysis was subsequently conducted to pinpoint metabolites that were significantly altered across the experimental groups. In total, 36 metabolites satisfied the selection thresholds of VIP > 1, p-value < 0.05, and fold change (FC) > 2.0 or < 0.5, and these were displayed in a heatmap after data normalization (Fig 12). The accompanying dendrogram showed that the Gul-BuOH–treated groups grouped closely with the normal controls and were clearly separated from the diseased group, suggesting that Gul-BuOH treatment drove many of the disrupted metabolites back toward their normal physiological levels.

Comprehensive details for these differential metabolites, including retention times, molecular weights, molecular formulas, Human Metabolome Database (HMDB) identifiers, p-values, VIP scores, and their fold change and trends from normal to diseased (N–D) and from diseased to treated (D–T), are summarized in S1 Table. Relative to the healthy control group, the liver damage model showed reduced levels of 23 metabolites, namely, L-alanine, 2-methylalanine, butyric acid, dithioerythritol, heptenoic acid, glycerol, trimethylphosphate, L-proline, L-serine, L-threonine, arabinofuranose, *D*-ribose, phosphoric acid, 1,2,3-propanetricarboxylic acid, α-*D*-mannopyranose, methyl α-*D*-galactopyranoside, 5-alpha-cholest-7-en-3-one, gulonic acid, *D*-glucopyranose, palmitic acid, myo-inositol, oleic acid and cholesterol. Conversely, the levels of 13 metabolites namely, acetic acid, lactic acid, glycine, L-valine, urea, L-leucine, harmaline, L-pipecolic acid, trans-13,14-dihydroretinol, L-tyrosine, linoleic acid, stearic acid, and arachidonic acid were significantly increased in the diseased group. Remarkably, treatment with Gul-BuOH led to a substantial recovery in the levels of all the previously reduced metabolites, while the elevated level of most metabolites showed significant reduction, approaching levels seen in the healthy group. These findings suggest that Gul-BuOH may exert therapeutic effects by normalizing disrupted metabolic pathways associated with hepatotoxicity (S1 Table and Fig 12).

## 4. Discussion

Cadmium (Cd) is a highly toxic heavy metal known to induce severe hepatic injury in both humans and animals. Although numerous studies have demonstrated its capacity to damage liver cells, the precise mechanisms underlying Cd-induced hepatotoxicity remain incompletely understood [55]. The present study demonstrated the antioxidant and anti-inflammatory potential of Gul-BuOH in mitigating CdCl₂-induced hepatotoxicity. The HOTAIR/Let-7a axis represents the upstream regulatory epigenetic network disrupted by cadmium exposure, whereas the Sirt1/HO-1 axis reflects the downstream cyto-protective antioxidant defense system that becomes impaired as a consequence [55]. The combined application of qRT-PCR and immunohistochemistry enabled comprehensive tracking of this toxic cascade, from early transcriptional and epigenetic alterations to the resulting localized histopathological damage within hepatic tissue.

The findings provide valuable insights into the molecular mechanisms of Cd-induced liver injury, highlighting the modulation of SIRT1/TNF-α/NF-κB signaling and the regulation of circadian clock–associated miRNAs, thereby suggesting a promising protective role of Gul-BuOH against hepatic toxicity. CdCl_2_-induced hepatotoxicity is closely associated with enhanced lipid peroxidation and oxidative stress resulting from the excessive generation of reactive oxygen species (ROS). These highly reactive electrophilic molecules readily interact with cellular macromolecules, causing lipid peroxidative damage [56]. In addition, elevated levels of NOx and its reactive nitrogen species have been implicated in indiscriminate cellular injury during CdCl_2_-induced hepatotoxicity [57]. In agreement, our findings demonstrated marked oxidative damage following CdCl_2_ administration, as evidenced by significant elevations in MDA and NO, along with a concomitant reduction in TAC. Importantly, Gul-BuOH treatment effectively enhanced antioxidant defenses while attenuating lipid peroxidation products. Collectively, these results suggest that Gul-BuOH confers hepato-protection by reinforcing antioxidant mechanisms and suppressing oxidative stress–mediated lipid peroxidation.

Serum transaminases AST and ALT and alkaline phosphatase ALP are key indicators of hepatic cellular integrity, as elevated levels reflect increased membrane permeability and subsequent enzyme leakage into the bloodstream [58]. In the present study, CdCl_2_ administration significantly impaired liver function, as evidenced by elevated serum transaminases and corroborated by histopathological deterioration of hepatic tissues. These findings are consistent with previous reports documenting the hepatotoxic effects of CdCl_2_ [59]. In contrast, treatment with Gul-BuOH markedly restored serum hepatic markers to near-normal levels and ameliorated histopathological alterations, suggesting a protective effect against CdCl_2_-induced liver injury. These promising outcomes provided the rationale for further investigation into the molecular mechanisms underlying the hepatoprotective activity of Gul-BuOH.

SIRT1, a member of the sirtuin family, is an epigenetic regulator involved in multiple physiological and pathological processes, including inflammation [60]. It exhibits an inverse relationship with NF-κB, where reduced SIRT1 expression promotes NF-κB activation and exacerbates oxidative and inflammatory responses [61]. In the present study, CdCl₂ exposure markedly down regulated SIRT1 expression, consistent with previous reports linking its suppression to hepatic injury [62]. Notably, Gul-BuOH treatment significantly enhanced SIRT1 expression, as confirmed by immune-histochemical evaluation, suggesting that its hepatoprotective and anti-inflammatory effects may be mediated, at least in part, through SIRT1 up regulation.

Oxidative stress acts as a key trigger for inflammatory signaling in CdCl_2_-induced hepatic injury [59]. In the present study, CdCl_2_ exposure significantly up regulated NF-κB p65 activation and TNF-α expression, confirming the oxidative-inflammatory cascade previously reported in hepatic lesions [63]. NF-κB is a central mediator of CdCl₂-induced inflammation, promoting the overproduction of pro-inflammatory cytokines, including TNF-α [64], which further amplifies the response by stimulating downstream mediators such as NO [65]. In contrast, Gul-BuOH pre-treatment markedly it inhibit NF-κB p65 translocation and suppressed its activation and reduced TNF-α expression, indicating its ability to modulate NF-κB/TNF-α signaling. These findings suggest that the anti-inflammatory and hepatoprotective effects of Gul-BuOH are, at least in part, mediated through inhibition of the NF-κB/TNF-α pathway.

HO-1 plays a critical cyto-protective role by mitigating oxidative damage through the degradation of heme into carbon monoxide (CO), biliverdin/bilirubin, and free iron. These byproducts, particularly CO and biliverdin, have been shown to mediate the antioxidant, anti-inflammatory, and anti-apoptotic effects of HO-1 [66]. Deficiency of HO-1 is associated with severe oxidative stress and endothelial injury, whereas it’s up regulation is considered an adaptive response aimed at minimizing cellular damage [67]. Consistent with previous reports, our study demonstrated a marked suppression of HO-1 expression following CdCl_2_ administration, highlighting its involvement in CdCl_2_-induced hepatotoxicity [57]. Notably, treatment with Gul-BuOH significantly enhanced HO-1 expression, suggesting that its hepatoprotective effects may, at least in part, be mediated through activation of the HO-1 pathway.

MicroRNAs and lncRNAs have emerged as promising biomarkers, showing disease-specific expression patterns with strong diagnostic and prognostic value. Their stability in circulation further enhances their utility for detecting and monitoring pathological conditions [68]. In this study, exposure to CdCl_2_ exhibited cytotoxic effects, as evidenced by the marked down regulation of miR-let7a expression following treatment [69]. The decreased expression of miR-let7a may indicate a higher susceptibility of liver cells to carcinogenesis, given that the miR-let7 family normally suppresses let-60/RAS signaling in cancer [70]. Many studies reported a down regulation of miR-let7a prior to Cd exposure as an early event in the carcinogenesis process [71]. Moreover, members of the miR-let7 family are consistently down regulated in various cancers, while restoring their expression has been shown to inhibit tumor formation. These microRNAs also indirectly influence the cell cycle, differentiation, and apoptosis by modulating multiple transcription factors. Additionally, miR-let7 enhances the radio-sensitivity of several cell types [72]. Gul-BuOH produced a significant, dose-dependent up regulation of miR-let7a expression, suggesting a potential hepatoprotective effect, as it appears capable of modulating anti-tumor markers such as miR-let7a. HOTAIR lncRNA controls the epigenetic modification of numerous genes and plays a role in drug resistance, metastasis as a well-established oncogene with an essential function in different types of cancer, promoting their growth and progression its reduction markedly inhibited the proliferation and invasion of liver cancer cells [73]. This study showed that lncRNA HOTAIR expressions were significantly elevated in CdCl_2_ rats versus healthy ones. In line with this, HOTAIR were overexpressed when compared to control in metal toxicity, and these levels were well-matched with the progress of liver toxicity [74]. Gul-BuOH produced a significant, dose-dependent down regulation of lncRNA HOTAIR expression, suggesting a potential hepatoprotective effect, as it appears capable of modulating anti-tumor markers such as lncRNA HOTAIR.

Evaluating downstream of biochemical markers of hepatic injury and oxidative stress confirmed that observed changes in the expression of Sirt1/HO-1 and mi RNA Let7a and Inc RNA HOTAIR. They prove the preservation of hepatocyte membrane integrity by suppressed serum ALT, AST and ALP levels) and the mitigation of oxidative damage, by the functional restoration of cellular GSH, elevated TAC, and the suppression of MDA-mediated lipid peroxidation. Together, these biochemical endpoints functionally validate the molecular mechanisms proposed in this study.

### 4.1 Pathway enrichment analysis of differential metabolites

The differential biomarkers identified earlier were subsequently examined using the MetaboAnalyst 6.0 platform for metabolic pathway enrichment analysis (Fig 12). The results further highlight the strong link between hepatotoxicity development and its treatment with Gul-BuOH, as well as the associated biochemical mechanisms. Four major metabolic pathways were implicated: linoleic acid metabolism; phenylalanine, tyrosine and tryptophan biosynthesis; glycine, serine, and threonine metabolism; and ascorbate and aldarate metabolism. These pathways are explored in detail in the following subsections.

#### 4.1.1 Linoleic acid metabolism.

Recent studies indicate that linoleic acid (LA), beyond its role as an essential dietary fatty acid, can exert a hepatoprotective effect under conditions of induced liver injury. For instance, in a murine model of acute liver injury induced by lipopolysaccharide (LPS), LA supplementation significantly mitigated hepatic damage, lowered plasma markers of liver injury (ALT, AST, GST), and reduced pro-inflammatory mediators (TNF-α, IL-6, MPO) compared with untreated controls [75]. Mechanistically, this protective effect was associated with activation of the antioxidant defense pathway mediated by Nrf2 and upregulation of its downstream target NQO1, leading to enhanced levels of endogenous antioxidants (SOD, GSH, CAT, GSH-PX) and reduced lipid peroxidation (MDA) in liver tissue [75]. In a human epidemiological context, higher dietary intake of LA (as part of overall unsaturated fatty acids) was inversely associated with the risk of significant liver fibrosis, suggesting a potential protective role of LA metabolism in chronic liver disease [76]. Together, these findings support that LA metabolism may attenuate liver toxicity by modulating oxidative stress and inflammation, and by preserving hepatic structural integrity under toxic insults.

#### 4.1.2 Phenylalanine, tyrosine and tryptophan biosynthesis.

Recent evidence suggests that the metabolism of aromatic amino acids (AAAs), particularly Phenylalanine, tyrosine and tryptophan biosynthesis, may influence susceptibility to liver injury and represent a relevant metabolic axis in hepatoprotection. In a model of toxin-induced hepatic sinusoidal obstruction syndrome, supplementation with microbial tryptophan-derived metabolites (e.g., indole-3-acetaldehyde, indole-3-acetic acid) attenuated liver injury, reduced serum ALT/AST, decreased necrosis, and restored hepatic antioxidant balance *via* activation of the AhR/Nrf2 signaling pathway, implying that proper tryptophan metabolism and downstream microbial-derived products can protect against oxidative endothelial and hepatocyte damage [77]. Similarly, dietary supplementation of AAAs in mice improved lipid handling in the liver, decreasing hepatic and serum triglycerides and ameliorating steatosis; this effect was linked to upregulation of hepatic bile acid synthesis through enhanced expression of bile acid synthesis genes (e.g., Cyp7b1), suggesting AAAs may improve metabolic resilience of the liver under stress [78]. However, dysregulation in AAA metabolism appears in toxin- or drug-induced hepatotoxicity: in an animal model of chronic administration of a hepatotoxic drug Doxorubicin (DOX), hepatic levels of phenylalanine and tyrosine (and to a lesser extent tryptophan) dropped significantly and correlated inversely with serum aminotransferases (ALT/AST), indicating that disturbance of AAA homeostasis may reflect or contribute to liver injury [79]. Altogether, while the evidence is still emerging and context-dependent, these findings underscore a potential protective role for balanced AAA biosynthesis and metabolism, particularly tryptophan-derived microbial metabolites, in mitigating induced liver toxicity and suggest that modulation of AAA metabolic pathways might represent a therapeutic or preventive avenue in hepatotoxic settings.

#### 4.1.3 Glycine, serine and threonine metabolism pathway.

Recent evidence supports a hepatoprotective role of glycine and related amino acids via the Gly-Ser-Thr metabolic pathway in various models of liver injury. For instance, glycine supplementation mitigated high-fructose–induced hepatic steatosis and injury in adolescent mice, significantly reducing lipid accumulation, inflammation, oxidative stress, and hepatocyte apoptosis, while enhancing antioxidant defenses and promoting fatty-acid β-oxidation [80]. In a model of cholestasis (bile-duct ligation), glycine improved mitochondrial function in hepatocytes, restoring mitochondrial membrane potential and ATP production, reducing reactive oxygen species (ROS) and lipid peroxidation, thereby attenuating liver damage [81]. Moreover, in livers with steatosis (early stage of fatty liver disease), disrupted glycine availability (due to enhanced conversion to serine via reverse SHMT2 activity) impaired de novo glutathione (GSH) synthesis, sensitizing to xenobiotic-induced hepatotoxicity (e.g., by acetaminophen); glycine supplementation or SHMT2 ablation restored GSH synthesis and protected against acute toxicity [82]. Complementing these mechanistic insights, supplementation of serine, another component of this metabolic pathway, was shown to counteract oxidative stress in hepatocytes by supporting GSH synthesis and maintaining the methionine cycle, thereby reducing reactive oxygen species and cellular damage [82]. Altogether, these findings indicate that preservation of the Gly-Ser-Thr metabolic pathway, especially adequate glycine and serine availability, supports antioxidant capacity, mitochondrial integrity, and lipid homeostasis, thereby conferring resilience against induced liver toxicity and metabolic stress.

#### 4.1.4 Ascorbate and aldarate metabolism.

Recent evidence indicates that the ascorbate and aldarate metabolism pathway plays a key hepatoprotective role under toxic stress, primarily via the antioxidant and anti-inflammatory actions of ascorbic acid. In several experimental models of chemically induced liver injury (e.g., toxicants, pesticides, drugs), ascorbic acid supplementation reduced elevations of serum liver injury markers (ALT, AST), mitigated lipid peroxidation (MDA), restored endogenous antioxidant defenses (e.g., GSH, SOD, CAT), and preserved histological integrity of liver tissue compared to untreated controls. For example, co-treatment with ascorbic acid significantly ameliorated hepatic oxidative stress and restored antioxidant enzyme activities in rat livers exposed to arsenic, demonstrating reduced lipid peroxidation and normalized glutathione and catalase levels [83]. In a model of drug-induced hepatic injury (e.g., with an antibiotic), ascorbic acid administration lowered inflammation (decreased pro-inflammatory cytokines), reduced oxidative stress (lower MDA, nitric oxide), increased catalase and GSH, and improved histopathological outcomes [84]. Mechanistically, such protection likely reflects ascorbate’s role in scavenging reactive oxygen species, stabilizing hepatic mitochondrial and cellular membranes, inhibiting lipid peroxidation, and maintaining redox balance — processes central to the ascorbate/aldarate metabolic pathway as defined in metabolic pathway analyses [85]. Altogether, these observations support the concept that intact or supplemented ascorbate/aldarate metabolism can attenuate toxin-induced hepatic injury, preserving liver function and structural integrity under oxidative and inflammatory challenge.

The hepatoprotective effect of Gul-BuOH’s may be attributed to the complementary and potentially synergistic effects of its chemically varied metabolite profile, rather than a single component.. Flavonoids seem to be the main bioactive contributors at the individual level, especially methoxylated flavonols, flavanones, flavan-3-ols, and flavone *C*-glycosides. By suppressing lipogenic and fibrogenic pathways, isorhamnetin and quercetin glycosides have potent antioxidant and anti-fibrotic actions [86,87]. This is consistent with the reported decrease in oxidative stress markers (MDA, NO), normalization of liver enzymes, and histological healing. In line with the significant downregulation of NF-κB-p and TNF-α and the restoration of HO-1 expression, procyanidin oligomers further strengthen this defense by scavenging reactive oxygen species and blocking inflammatory signaling cascades like NF-κB and MAPK [78].

Phenolic acids—which are primarily hydroxybenzoates and derivatives of caffeic acid—likely work in concert with flavonoids to restore TAC and lessen metabolic disruptions brought on by cadmium by increasing the overall antioxidant buffering capacity and reducing lipid peroxidation [88]. Vicenin-2 and other flavone *C*-glycosides are especially important because of their greater metabolic stability, which allows for long-lasting intracellular anti-inflammatory and antioxidant actions that may promote long-term hepatocellular resilience [89,90]. Beyond polyphenols, lipid-derived metabolites play a significant role. The enhancement in Sirt-1 expression and the normalization of lipid-related metabolic pathways seen in serum metabolomics may be explained by the modulation of inflammatory tone and cellular signaling by oxygenated fatty acids and fatty acyl amides [91], which are described here for the first time in *G. ulmifolia*. Additionally, these lipid mediators may help stabilize membranes and reduce the buildup of cadmium in hepatic tissue [91]. In the meantime, organic acids like succinic and malic acids may promote redox equilibrium and mitochondrial metabolism, so indirectly supporting energy balance and hepatocyte survival in the face of toxic stress [92]. The convergence of these compound classes causes a multi-targeted response at the systems level, including the reduction of oxidative stress and inflammation, the improvement of endogenous cytoprotective pathways (HO-1 and Sirt-1), the control of non-coding RNAs (upregulation of miRNA Let-7a and downregulation of lncRNA HOTAIR), and the restoration of disturbed metabolism of amino acids, fatty acids, and carbohydrates [93]. This comprehensive approach is supported by the serum metabolomics and pathway enrichment analyses, which demonstrate the recovery of important pathways linked to redox balance, amino acid biosynthesis, and lipid metabolism. All of these results point to a synergistic phytochemical network in Gul-BuOH, where bioactive lipids, phenolic acids, and flavonoids work together to prevent hepatotoxicity caused by cadmium and restore hepatic homeostasis.

This study’s primary goal was to characterize the independent mechanistic and functional effects of *G. ulmifolia* leaf extract in a cadmium-induced toxicological model. However, the study did not include a comparative evaluation against well-known reference hepatoprotective agents like silymarin, which could have offered more efficacy benchmarking. Additionally, dose-optimization studies and comparative phytochemical analyses of various plant parts were not carried out; these are necessary in subsequent research to determine the most efficient and consistent treatment plan. A thorough dose-response analysis was not carried out, despite the fact that two treatment dosages (100 and 200 mg/kg) were assessed and the extract had previously been shown to be safe. Therefore, additional study is needed to determine the ideal therapeutic dose and dosing schedule.

Lastly, the results were only produced in a rat model that was exposed to cadmium. The complex nature of human liver illnesses or long-term environmental cadmium exposure is not adequately represented by this model, despite the fact that it is well-established for examining heavy metal-induced hepatotoxicity. Therefore, it is important to be cautious when applying these findings directly to clinical scenarios. Standardized leaf extracts, pharmacokinetic analyses, optimal dose plans, comparative efficacy studies with proven hepatoprotective drugs, validation in more preclinical models, and clinical research should all be included in future studies. By addressing these issues, the findings’ generalizability and translational significance will be enhanced, and the therapeutic potential of *G. ulmifolia* leaf extract as a multi-target hepatoprotective drug will be further defined.

### Conclusion

The present investigation suggests that the *G. ulmifolia* exerts a dose-dependent hepatoprotective effect against cadmium-induced liver injury in vivo. by lowering the hepatic cadmium burden, reestablishing the antioxidant balance, reducing inflammation, and restoring normal liver function. A rich and uncharted phytochemical landscape was uncovered by thorough UPLC-MS-based analysis. Notably, *G. ulmifolia* also reprogrammed significant molecular and metabolic anomalies, such as disrupted lipid and amino acid pathways and non-coding RNA regulation, to return the damaged liver to a healthy state. When considered collectively, these integrated phytochemical-metabolomic insights provide compelling scientific evidence for the traditional use of *G. ulmifolia* and demonstrate its efficacy as a natural, systems-level therapeutic strategy for heavy metal-associated liver damage.

## Supporting information

S1 Fig ESI-MS/MS spectrum of peak 33 in the negative ion mode. (DOCX)

S2 Fig ESI-MS/MS spectrum of peak 16 in the negative ion mode. (DOCX)

S3 Fig ESI-MS/MS spectrum of peak 23 in the negative ion mode. (DOCX)

S4 Fig ESI-MS/MS spectrum of peak 11 in the negative ion mode. (DOCX)

S5 Fig ESI-MS/MS spectrum of peak 6 in the negative ion mode. (DOCX)

S6 Fig ESI-MS/MS spectrum of peak 4 in the negative ion mode. (DOCX)

S7 Fig ESI-MS/MS spectrum of peak 47 in the positive ion mode. (DOCX)

S8 Fig Dendrogram-heatmap displaying serum metabolic profiles of normal, diseased and after Gul-BuOH treatment groups. (DOCX)

S9 Fig Permutation plots for OPLS-DA model (a) normal, (b) cadmium chloride-induced hepatotoxicity and (c) Gul-BuOH- treated groups using 20 permutations. (DOCX)

S1 Table Key differential metabolites of serum samples of normal (N), hepatotoxicity-diseased (D) and G. ulmifolia butanol extract-treated (T) groups. (DOCX)

S1 File Raw data file of MIRlet7a, LNC HOTAIR, NF-KB, TNF-α, ALT, AST, ALP, TAC, MDA, NO, and Cd concentration. (DOCX)
