# Supplementary material for: Guazuma ulmifolia butanol extract protects against cadmium-induced hepatotoxicity via HO-1/Sirt-1 activation, miRNA–lncRNA modulation, and metabolic reprogramming
Source: PLoS One. 2026 Aug 6;21(8):e0355464. doi: 10.1371/journal.pone.0355464 (PMC13446703; doi:10.1371/journal.pone.0355464)
Supplement: Supplementary file 9 [file pone.0355464.s009.docx]

**Suppl. Fig. S9:** Permutation plots for OPLS-DA model (a) normal, (b) cadmium chloride-induced hepatotoxicity and (c) Gul-BuOH- treated groups using 20 permutations.
