# Supplementary material for: Guazuma ulmifolia butanol extract protects against cadmium-induced hepatotoxicity via HO-1/Sirt-1 activation, miRNA–lncRNA modulation, and metabolic reprogramming
Source: PLoS One. 2026 Aug 6;21(8):e0355464. doi: 10.1371/journal.pone.0355464 (PMC13446703; doi:10.1371/journal.pone.0355464)
Supplement: Supplementary file 10 [file pone.0355464.s010.docx]

**Table 1S:** Key differential metabolites of serum samples of normal (N), hepatotoxicity-diseased (D) and *G. ulmifolia* butanol extract-treated (T) groups

| **No.** | **T_R_ (min.)** | **Metabolites** | **Molecular weight** | **Molecular formula** | **HMDB ID** | **p-Value** | **VIP** | **FC Trend  (D-N)** | **FC Trend  (T-D)** |
| --- | --- | --- | --- | --- | --- | --- | --- | --- | --- |
| 1 | 6.429 | Acetic acid | 60.0520 | C_2_H_4_O_2_ | HMDB0000042 | 4.6457E-16 | 1.1329 | 0.2702 (↓) | 0.1719 (↓) |
| 2 | 6.456 | Lactic acid | 90.0779 | C_3_H_6_O_3_ | HMDB0000190 | 2.9912E-16 | 1.04131 | 0.2040 (↓) | 0.2919 (↓) |
| 3 | 7.500 | L-Alanine | 89.0932 | C_3_H_7_NO_2_ | HMDB0000161 | 1.4598E-14 | 1.13221 | 3.0390 (↑) | 0.2492 (↓) |
| 4 | 7.548 | Glycine | 75.0666 | C_2_H_5_NO_2_ | HMDB0000123 | 1.5605E-15 | 1.13364 | 0.3110 (↓) | 0.3538 (↓) |
| 5 | 8.482 | 2-Methylalanine | 103.1198 | C_4_H_9_NO_2_ | HMDB0001906 | 1.1542E-15 | 1.13221 | 4.3605 (↑) | 0.1513 (↓) |
| 6 | 8.572 | Butyric acid | 88.1051 | C_4_H_8_O_2_ | HMDB0000039 | 1.6642E-15 | 1.12832 | 3.0457 (↑) | 0.1534 (↓) |
| 7 | 8.922 | Dithioerythritol | 154.2400 | C_4_H_10_O_2_S_2_ | HMDB0251493 | 1.5568E-15 | 1.1297 | 2.1790 (↑) | 0.0262 (↓) |
| 8 | 9.279 | Heptenoic acid | 128.1690 | C_7_H_12_O_2_ | HMDB0033793 | 8.6029E-16 | 1.04919 | 4.7901 (↑) | 0.1456 (↓) |
| 9 | 9.800 | L-Valine | 117.1463 | C_5_H_11_NO_2_ | HMDB0000883 | 1.4578E-16 | 1.10499 | 0.1643 (↓) | 0.1575 (↓) |
| 10 | 10.173 | Urea | 60.0553 | CH_4_N_2_O | HMDB0000294 | 2.2606E-15 | 1.13261 | 0.4134 (↓) | 0.1768 (↓) |
| 11 | 10.405 | L-Leucine | 131.1729 | C_6_H_13_NO_2_ | HMDB0000687 | 2.6434E-12 | 1.01496 | 0.3167 (↓) | 2.4032 (↑) |
| 12 | 10.459 | Harmaline | 214.2631 | C_13_H_14_N_2_O | HMDB0030310 | 1.4531E-15 | 1.12687 | 0.0145 (↓) | 32.679 (↑) |
| 13 | 10.827 | Glycerol | 92.0938 | C_3_H_8_O_3_ | HMDB0000131 | 4.7596E-16 | 1.11684 | 4.1130 (↑) | 0.0683 (↓) |
| 14 | 10.877 | Trimethylphosphate | 140.0749 | C_3_H_9_O_4_P | HMDB0259237 | 1.1849E-15 | 1.13098 | 2.1015 (↑) | 0.0003 (↓) |
| 15 | 11.337 | L-Proline | 115.1305 | C_5_H_9_NO_2_ | HMDB0000162 | 1.8065E-15 | 1.1318 | 2.9619 (↑) | 0.1024 (↓) |
| 16 | 12.479 | L-Serine | 105.0926 | C_3_H_7_NO_3_ | HMDB0000187 | 1.2675E-14 | 1.13112 | 2.2381 (↑) | 0.1621 (↓) |
| 17 | 13.234 | L-Threonine | 119.1192 | C_4_H_9_NO_3_ | HMDB0000167 | 8.4578E-15 | 1.10515 | 2.0078 (↑) | 0.1144 (↓) |
| 18 | 15.517 | L-Pipecolic acid | 129.1570 | C_6_H_11_NO_2_ | HMDB0000716 | 2.2397E-15 | 1.06112 | 0.1429 (↓) | 2.1097 (↑) |
| 19 | 16.343 | Trans-13,14-dihydroretinol | 288.4675 | C_20_H_32_O | HMDB0011618 | 6.7981E-17 | 1.10008 | 0.0933 (↓) | 0.0908 (↓) |
| 20 | 17.968 | Arabinofuranose | 150.1299 | C_5_H_10_O_5_ | HMDB0012325 | 2.6264E-14 | 1.13324 | 3.3210 (↑) | 0.3150 (↓) |
| 21 | 19.579 | D-Ribose | 150.1299 | C_5_H_10_O_5_ | HMDB0000283 | 2.7991E-15 | 1.12826 | 2.5144 (↑) | 0.0957 (↓) |
| 22 | 20.671 | Phosphoric acid | 97.9952 | H_3_O_4_P | HMDB0001429 | 4.5196E-15 | 1.1322 | 3.3456 (↑) | 0.1945 (↓) |
| 23 | 21.850 | 1,2,3-Propanetricarboxylic acid | 176.1241 | C_6_H_8_O_6_ | HMDB0031193 | 9.1322E-15 | 1.13347 | 3.5639 (↑) | 0.2605 (↓) |
| 24 | 21.977 | alpha-D-Mannopyranose | 180.1559 | C_6_H_12_O_6_ | HMDB0000169 | 6.7406E-15 | 1.13198 | 2.3420 (↑) | 0.1350 (↓) |
| 25 | 22.564 | L-Tyrosine | 181.1885 | C_9_H_11_NO_3_ | HMDB0000158 | 7.674E-15 | 1.13233 | 0.2861 (↓) | 7.7220 (↑) |
| 26 | 22.800 | Methyl alpha-D-galactopyranoside | 194.1825 | C_7_H_14_O_6_ | HMDB0246173 | 1.8223E-16 | 1.13236 | 11.268 (↑) | 0.1185 (↓) |
| 27 | 23.476 | 5alpha-Cholest-7-en-3-one | 384.6377 | C_27_H_44_O | HMDB0034399 | 3.9227E-17 | 1.13122 | 57.903 (↑) | 0.0153 (↓) |
| 28 | 23.781 | Gulonic acid | 196.1553 | C_6_H_12_O_7_ | HMDB0003290 | 2.6434E-16 | 1.13067 | 5.0418 (↑) | 0.0532 (↓) |
| 29 | 24.231 | D-Glucopyranose | 180.1559 | C_6_H_12_O_6_ | HMDB0000122 | 6.1197E-13 | 1.13376 | 2.1433 (↑) | 0.3592 (↓) |
| 30 | 25.053 | Palmitic acid | 256.4241 | C_16_H_32_O_2_ | HMDB0000220 | 1.7518E-16 | 1.13139 | 9.8068 (↑) | 0.1010 (↓) |
| 31 | 26.161 | myo-Inositol | 180.1559 | C_6_H_12_O_6_ | HMDB0000211 | 1.5481E-15 | 1.1299 | 3.4400 (↑) | 0.1241 (↓) |
| 32 | 27.267 | Linoleic acid | 280.4455 | C_18_H_32_O_2_ | HMDB0000673 | 5.0781E-15 | 1.12612 | 0.4849 (↓) | 0.1851 (↓) |
| 33 | 27.686 | Oleic acid | 282.4614 | C_18_H_34_O_2_ | HMDB0000207 | 7.8794E-16 | 1.13083 | 4.4654 (↑) | 0.1251 (↓) |
| 34 | 27.871 | Stearic acid | 284.4772 | C_18_H_36_O_2_ | HMDB0000827 | 7.8321E-16 | 1.13301 | 0.3652 (↓) | 10.515 (↑) |
| 35 | 29.507 | Arachidonic acid | 304.4669 | C_20_H_32_O_2_ | HMDB0001043 | 1.6115E-13 | 1.04534 | 0.1940 (↓) | 4.1528 (↑) |
| 36 | 38.869 | Cholesterol | 386.6535 | C_27_H_46_O | HMDB0000067 | 1.5967E-16 | 1.13189 | 8.2209 (↑) | 0.0725 (↓) |

T_R_ (min.): retention time; N: normal group; D: diseased group; T: treated group; HMDB ID: human metabolome database identifier; VIP: variable importance in the projection; FC: fold change.
