# Supplementary material for: Guazuma ulmifolia butanol extract protects against cadmium-induced hepatotoxicity via HO-1/Sirt-1 activation, miRNA–lncRNA modulation, and metabolic reprogramming
Source: PLoS One. 2026 Aug 6;21(8):e0355464. doi: 10.1371/journal.pone.0355464 (PMC13446703; doi:10.1371/journal.pone.0355464)
Supplement: Supplementary file 11 [file pone.0355464.s011.docx]

**Raw data**

| **MIRlet7a** | | | |
| --- | --- | --- | --- |
| Normal control | CdCl2 | Gul-BuOH (100 mg/kg) | Gul-BuOH (200 mg/kg) |
| 1.01 | 0.26 | 0.75 | 0.81 |
| 1 | 0.27 | 0.78 | 0.84 |
| 1.02 | 0.22 | 0.79 | 0.79 |
| 1.03 | 0.23 | 0.74 | 0.82 |
| 1.04 | 0.24 | 0.76 | 0.83 |
| 1.05 | 0.25 | 0.77 | 0.8 |

| **LNC HOTAIR** | | | |
| --- | --- | --- | --- |
| Normal control | CdCl2 | Gul-BuOH (100 mg/kg) | Gul-BuOH (200 mg/kg) |
| 1 | 4.3 | 2.01 | 1.9 |
| 1.01 | 4.2 | 1.4 | 2.3 |
| 1 | 3.9 | 1.6 | 1.2 |
| 1.2 | 4 | 1.8 | 2.2 |
| 1.3 | 4.1 | 1.9 | 2.1 |
| 0.9 | 4.4 | 1.7 | 2 |

| **NF-KB** | | | |
| --- | --- | --- | --- |
| Normal control | CdCl2 | Gul-BuOH (100 mg/kg) | Gul-BuOH (200 mg/kg) |
| 0.86 | 6.1 | 2.2 | 1.09 |
| 0.72 | 5.1 | 2.8 | 1.37 |
| 1.12 | 5.31 | 2.6 | 1.2 |
| 0.99 | 5.5 | 1.9 | 1.1 |
| 1.23 | 5.71 | 1.8 | 1.45 |
| 1.03 | 5.94 | 1.78 | 1.12 |

| **TNF-α** | | | |
| --- | --- | --- | --- |
| Normal control | CdCl2 | Gul-BuOH (100 mg/kg) | Gul-BuOH (200 mg/kg) |
| 43.3 | 157.8 | 61.5 | 58.2 |
| 49.7 | 155.2 | 58.2 | 56.3 |
| 45.5 | 153.1 | 60 | 54.5 |
| 47.3 | 151.3 | 63.2 | 52.1 |
| 51.2 | 149.2 | 65.4 | 50.3 |
| 47.4 | 153.32 | 61.66 | 54.28 |

| **ALT** | | | |
| --- | --- | --- | --- |
| Normal control | CdCl2 | Gul-BuOH (100 mg/kg) | Gul-BuOH (200 mg/kg) |
| 12 | 21 | 12 | 11 |
| 12 | 25 | 17 | 5 |
| 11 | 29 | 15 | 7 |
| 10 | 32 | 11 | 8 |
| 9 | 32 | 14 | 10 |
| 11 | 27 | 16 | 9 |

| **AST** | | | |
| --- | --- | --- | --- |
| Normal control | CdCl2 | Gul-BuOH (100 mg/kg) | Gul-BuOH (200 mg/kg) |
| 36 | 67 | 47 | 47 |
| 37 | 68 | 48 | 46 |
| 37 | 69 | 49 | 45 |
| 38 | 70 | 50 | 43 |
| 39 | 71 | 51 | 42 |
| 41 | 72 | 52 | 41 |

| **ALP** | | | |
| --- | --- | --- | --- |
| Normal control | CdCl2 | Gul-BuOH (100 mg/kg) | Gul-BuOH (200 mg/kg) |
| 98.32812 | 152.4537 | 101.0344 | 92.01347 |
| 93.81766 | 153.3558 | 115.4679 | 96.52394 |
| 99.23021 | 165.9851 | 138.9223 | 101.0344 |
| 108.2511 | 186.7332 | 120.8804 | 106.447 |
| 110.0553 | 154.2579 | 130.8035 | 109.1532 |
| 106.447 | 169.5935 | 107.349 | 116.37 |

| **TAC** | | | |
| --- | --- | --- | --- |
| Normal control | CdCl2 | Gul-BuOH (100 mg/kg) | Gul-BuOH (200 mg/kg) |
| 0.469 | 0.281 | 0.371 | 0.423 |
| 0.501 | 0.22 | 0.3312 | 0.481 |
| 0.513 | 0.261 | 0.318 | 0.422 |
| 0.487 | 0.243 | 0.351 | 0.461 |
| 0.491 | 0.211 | 0.311 | 0.441 |
| 0.4922 | 0.2432 | 0.33644 | 0.4456 |

| **MDA** | | | |
| --- | --- | --- | --- |
| Normal control | CdCl2 | Gul-BuOH (100 mg/kg) | Gul-BuOH (200 mg/kg) |
| 4.84 | 10 | 5.51 | 4.61 |
| 4.72 | 9.8 | 5.1 | 4.52 |
| 4.62 | 9.71 | 5.2 | 4.43 |
| 4.51 | 9.61 | 5.31 | 4.31 |
| 4.45 | 9.52 | 5.42 | 4.23 |
| 4.628 | 9.728 | 5.308 | 4.42 |

| **NO** | | | |
| --- | --- | --- | --- |
| Normal control | CdCl2 | Gul-BuOH (100 mg/kg) | Gul-BuOH (200 mg/kg) |
| 0.247 | 0.631 | 0.251 | 0.209 |
| 0.268 | 0.606 | 0.291 | 0.213 |
| 0.273 | 0.624 | 0.283 | 0.223 |
| 0.252 | 0.617 | 0.276 | 0.248 |
| 0.231 | 0.645 | 0.263 | 0.25 |
| 0.2542 | 0.6246 | 0.2728 | 0.2286 |

| **Cd Con.** | | | |
| --- | --- | --- | --- |
| Normal control | CdCl2 | Gul-BuOH (100 mg/kg) | Gul-BuOH (200 mg/kg) |
| 0 | 21.372 | 7.68 | 4.67 |
| 0 | 15.585 | 6.5 | 6.23 |
| 0 | 18.4785 | 6.1 | 5.1 |
| 0 | 16.9 | 7.1 | 5.7 |
| 0 | 19.3 | 8.2 | 6.5 |
| 0 | 22.2 | 8.7 | 6.9 |
