# Supplementary material for: Guazuma ulmifolia butanol extract protects against cadmium-induced hepatotoxicity via HO-1/Sirt-1 activation, miRNA–lncRNA modulation, and metabolic reprogramming
Source: PLoS One. 2026 Aug 6;21(8):e0355464. doi: 10.1371/journal.pone.0355464 (PMC13446703; doi:10.1371/journal.pone.0355464)
Supplement: Supplementary file 12 [file pone.0355464.s001.docx]

**Suppl. Fig. S1:** ESI-MS/MS spectrum of peak **33** in the negative ion mode.
